# Comparing the efficacy and safety of cisplatin and other platinum-based chemotherapies in locally advanced nasopharyngeal carcinoma: a systematic review and meta-analysis

**DOI:** 10.1186/s12885-022-09712-z

**Published:** 2022-06-06

**Authors:** Zhiru Li, Chao Li, Dong Yang, Junmei Song, Ting Liu, Ziyan Zhou, Lifang Zhou, Min Kang

**Affiliations:** 1 Department of Radiation Oncology, The First Affiliated Hospital of Guangxi Medical University, No. 6, Shuangyong Road, Nanning, 530021 Guangxi People’s Republic of China; 2 Guangxi Tumor Radiation Therapy Clinical Medical Research Center, Nanning, Guangxi People’s Republic of China; 3 Department of Oncology, Sichuan Provincial People’s Hospital·Qionglai Medical Center Hospital, Chengdu, Sichuan People’s Republic of China; 4 Department of Obstetrics and Gynecology, Sichuan Provincial People’s Hospital·Qionglai Medical Center Hospital, Chengdu, Sichuan People’s Republic of China; 5 The Fourth Affiliated Hospital of Guangxi Medical University, Liuzhou Center for Disease Prevention and Control, Liuzhou, Guangxi People’s Republic of China

**Keywords:** Cisplatin, Platinum-based derivatives, Survival, Side effect, NPC, Meta-analysis

## Abstract

**Background:**

Cisplatin-based concurrent chemoradiotherapy has been identified as the primary and standard treatment for locally advanced nasopharyngeal carcinoma (NPC). However, the side effects of cisplatin affect the compliance to therapy. Thus, the search for a platinum-based substitute for NPC has always been a research focus. However, there is a variability in the efficacy of different platinum-based chemotherapies in the treatment of NPC. We performed a meta-analysis to compare the efficacy and safety of cisplatin-based regimens and other platinum-based derivatives (carboplatin, nedaplatin, and lobaplatin) for locally advanced NPC.

**Methods:**

PubMed, EMBASE, Cochrane Library, Web of Science, and ClinicalTrials.gov were systematically searched for all potentially eligible clinical trials as of February 15, 2022. The pooled hazard ratios, risk ratio, and 95% confidence interval were calculated using Review Manager Software version 5.4.

**Results:**

A total of 1,907 patients with locally advanced NPC were eligible from the 1,265 retrieved records. This systematic review included eight articles, six of which were randomized controlled clinical trials. There was no significant difference in the 3- and 5-year overall survival, progression-free survival, distant metastasis-free survival, and locoregional relapse-free survival between cisplatin-based chemotherapy and other platinum-based chemotherapy. Severe acute hematological side effects (≥ grade 3) during treatment, such as neutropenia, leukopenia, and thrombocytopenia, were equivalent in both groups. However, the incidence of anemia was higher in patients receiving other platinum-based chemotherapies. The risk of nausea, vomiting and weight loss was higher in the cisplatin group; however, there was no significant difference in the other non-hematological and late side effects between the two groups.

**Conclusions:**

Other types of platinum-based chemotherapies are as effective as cisplatin-based chemotherapy in the treatment of locally advanced NPC, thus acting as potential alternatives to cisplatin. Further studies providing high-level evidence are needed.

**Supplementary Information:**

The online version contains supplementary material available at 10.1186/s12885-022-09712-z.

## Background

The global geographical distribution of nasopharyngeal carcinoma (NPC) is unbalanced, with > 70% of the new NPC cases being reported in China and Southeastern Asia. An age-standardized incidence rate between 3.0 and 10.2 per 100,000 people has been reported in China [1, 2]. More than 70% of newly diagnosed NPC cases are classified as locally advanced disease in stages II–IVB [3]. Cisplatin-based concurrent chemoradiotherapy (CCRT) has been identified as the primary and standard treatment for locally advanced NPC. Although cisplatin offers substantial survival benefits to patients [3–5], its limitations lie in the poor adherence to treatment and side effects such as nausea, vomiting, nephrotoxicity, ototoxicity, and neurotoxicity [6, 7]. Therefore, there is an emerging need for other chemotherapeutic agents with similar efficacy against NPC and fewer side effects. Other platinum-based derivatives such as nedaplatin, lobaplatin and carboplatin have similar efficacy and fewer side effects, thus they have been used to replace cisplatin in the treatment of NPC [8–10]. However, no statistically significant results have been obtained from these studies. Thus, the aim of this meta-analysis of published clinical trials, retrospective studies, and paired analyses, was to compare the efficacy and safety of cisplatin-based and other platinum-based regimens in the treatment of locally advanced NPC.

## Methods

### Search strategy

We conducted a thorough search of the databases of medical publications: PubMed trial, EMBASE, Cochrane Library, Web of Science, and ClinicalTrials.gov, searching for all available records until February 15, 2022. The search was conducted by "subject word" or "title or key word." The search terms included: “Nasopharyngeal carcinoma,” “Carcinoma, Nasopharyngeal,” “Carcinomas, Nasopharyngeal,” “Nasopharyngeal Carcinomas,” “Cisplatin,” “lobaplatin,” “Nedaplatin,” “carboplatin,” and “randomized controlled trial or Randomized or placebo or RCT.” We manually searched the references of relevant articles to retrieve more clinical studies. In addition, a search was conducted before the final analysis. Two researchers (ZL and CL) independently screened the literature from the above databases and selected articles that met the inclusion criteria by reading the title or abstracts. If published data overlapped, only the most current information was included. In addition, a third researcher (DY) intervened to resolve any dispute(s).

### Inclusion criteria

All the studies included in this meta-analysis followed the PICOS principles (Participants, Intervention, Comparison and Outcomes, Study design). The details are as follows: (1) P: patients with stage II–IVB locally advanced NPC diagnosed by pathology; (2) I: Patients in the experimental group received chemotherapy with other platinum derivatives (carboplatin, nedaplatin, and lobaplatin), while the control groups received cisplatin chemotherapy. The specific combination of chemotherapy and radiotherapy techniques were ignored in both groups; (3) C: analysis of therapeutic efficacy and toxicity during and after radiotherapy and chemotherapy; (4) O: major positive outcomes include overall survival (OS), progression-free survival (PFS), distant metastasis-free survival (DMFS), locoregional relapse-free survival (LRFS), while negative outcomes include hematologic and non-hematologic toxicities; (5) S: we not only included randomized controlled trials, but also observational studies (including cohort and case–control studies).

### Exclusion criteria

Studies with any of the following characteristics were excluded: (1) studies on recurrent and metastatic nasopharyngeal carcinoma, (2) studies including patients with prior treatment with immunosuppressants or antiangiogenic drugs, (3) studies lacking detailed information or conference summaries, (4) unpublished studies, (5) single-arm clinical trials.

### Data extraction and quality assessment

The following details were extracted from each eligible clinical trial: first author, publication year, inclusion period, registration number, study design, number of patients, tumor stage, mean age, median follow-up period, therapeutic regimens, OS, PFS, DMFS, LRFS, and adverse events.

Two assessment scales were used to assess the methodological quality of each eligible trial. The Cochrane risk bias assessment tool [11] was used to evaluate the quality of included randomized controlled clinical trials (RCTs). The quality evaluation included six aspects: random sequence generation, assignment hiding, blind method implementation, data integrity, reporting bias and other bias. There were three options for each: “low risk,” “high risk,” or “unclear.” The quality of the two retrospective studies was evaluated using the Newcastle–ottawa Scale (NOS) [12], including study population selection, intergroup comparability, and outcome measurements. It was graded by the semi-quantitative principle of the star system, the full score is 9, and ≥ 6 is classified as high-quality literature. The final NOS scale defined two retrospective studies as high-quality studies. The two researchers (ZL and CL) independently conducted and cross-checked the above-mentioned literature quality during the evaluation process. In case of any disagreement, the third researcher (DY) was consulted.

### Statistical analysis

Summary statistics were compiled using the Review Manager Software, version 5.4 (Cochrane Collaboration RevMan, version 5.4, Oxford, UK). Survival outcomes (OS, PFS, DMFS, and LRFS) were assessed by hazard ratios (HRs) and 95% confidence intervals (CIs). If the HR was not directly described in the paper, Engauge Digitalizer version 4.1 software was used to extract data from the Kaplan–Meier survival curves according to the method of Tierney et al. [13], then the natural logarithm of HR (InHR) and standard error could be calculated. The relative risk (RR) was used to quantify and analyze efficacy. The inverse variance (IV) method was used to evaluate HR, and the Mantel Haenszel method was used to evaluate RR. The Χ^2^ test and I^2^ statistical and quantitative heterogeneity tests were used in each study, where *p* < 0.10 or I ^2^> 50% indicated that there was heterogeneity in each study and the random effect model was used for analysis. However, *p* > 0.10 or I^2^ < 50% indicated no statistical heterogeneity (H) and the fixed effect model was used for analysis. Sensitivity analysis excluded any element from the study and observed its impact on the combined statistics and the heterogeneity of test results.

## Results

### Study selection

**Fig. 1:**
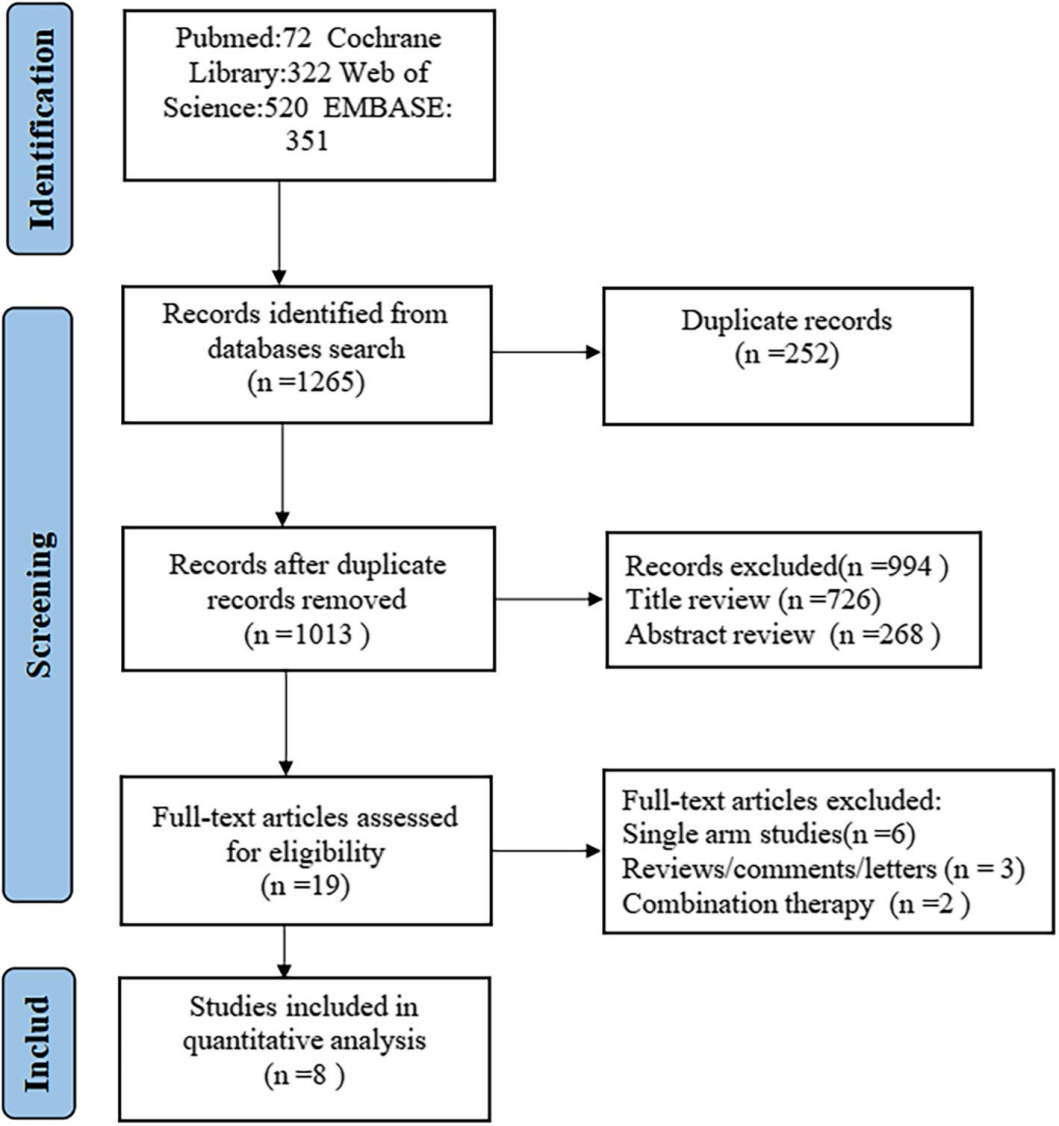
Flow chart of the study selection process

A total of 1,265 articles were retrieved from the PubMed, EMBASE, Cochrane Library and Web of Science databases. Two hundred and fifty-two duplicate records were deleted. After screening the title and abstract, there were 19 qualified articles left. After reading the full texts, eight studies [14–22] were finally included in the meta-analysis. The specific process of research screening is shown in Fig. 1.

### Eligible studies and characteristics

**Fig. 2:**
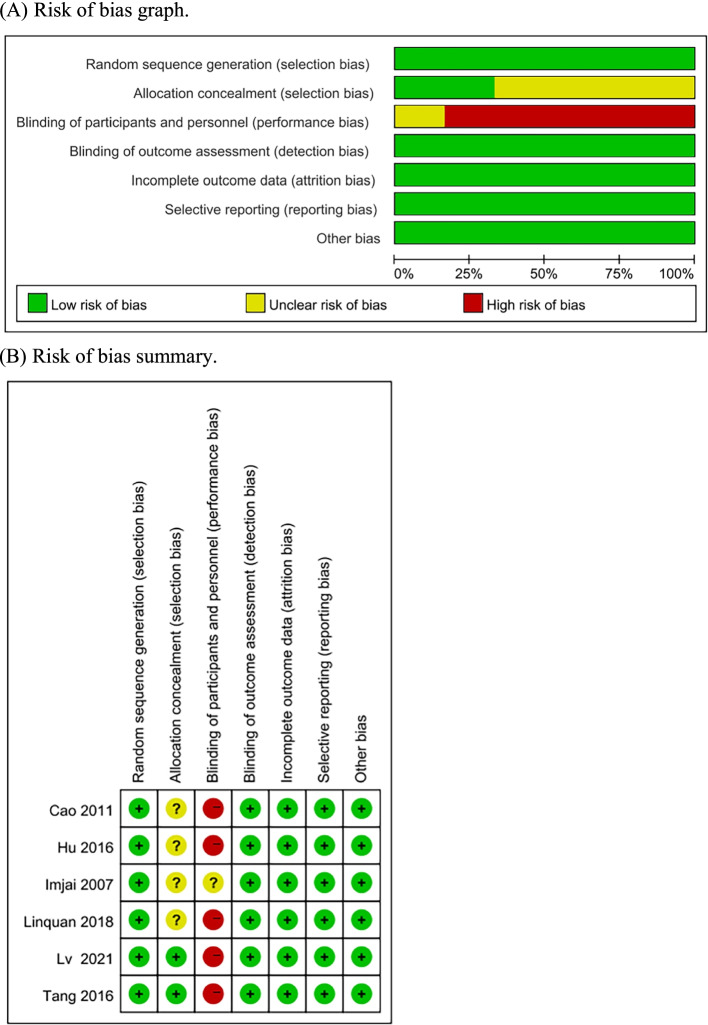
Risk of bias: Review authors’ judgments regarding bias in the RCTs included in this study

The eight studies included in this review included a total of 1,907 patients. Six of the eight studies were RCTs, while the other two were retrospective studies. Through the Cochrane bias risk analysis tool, four RCTs [14–18] were noted as having used a random number method and the other two RCTs [21, 22] did not indicate specific random methods. All RCTs included in this study did not explain hidden groups and there was no indication that blinding was applied to patients and doctors. However, most of the outcome indicators for those RCTs were based on clinical data, and the blinding method has a relatively little impact on the clinical data. All the literature data were complete, where no missing information or incomplete data affected the analysis of the results, and no selective reports or other sources of bias were found in the studies. The details about the risk bias are shown in Fig. 2. The NOS scale defined two retrospective studies as high-quality studies. Table 1 shows the basic characteristics of the eligible clinical trials, while Table 2 shows the details and outcome measures of the treatment regimens.

**Table 1:** Characteristics of the eligible studies

Study	Inclusion period	Register	Type of study	Phase	chemoradiotherapy	No.Patients	No.male	Mean Age (Exp/con)	AJCC Stage	Median follow-up(year)
Lv et al	2013–2015	ChiCTR-TRC-13003285	RCT	III	IC + CCRT	502	362	43.5/44	III–IVB	75.3
Tang et al	2011–2012	NCT 01479504	RCT	III	IC + CCRT	223	NR	45.1/45.3	III–IV	35.1
Linquan et al	2012–2014	NCT01540136	RCT	III	CCRT	402	302	44/45	II–IVB	47
Hu et al	2014–2015	NR	RCT	III	IC	62	45	50.2/49.8	III–IV	NR
Liu et al	2009–2011	NR	RE	FE	IC + CCRT	186	119	NR	II–IVB	68
Zhan et al	2012–2017	NR	RE	FE	IC + CCRT	226	184	NR	III–IVA	39.5
Cao et al	2009–2010	NR	RCT	III	IC	100	NR	NR	III–IVA	NR
Imjai et al	1999–2004	NR	RCT	III	CCRT + AC	206	126	50/46	III–IVB	26.3

*RCT* randomized controlled trials, *IC* Induction chemotherapy, *CCRT* Concurrent chemoradiotherapy, *AC* Adjuvant chemotherapy, *Con* control group (cisplatin-based group), *Exp* experimental group (other platinum-based group), *NA* not available, *Re* retrospetive study, *NR* not reported, *FE* fail to extract

**Table 2:** Therapeutic regimens, survival outcomes in eligible studies

Study	Induction chemotherapy	Concurrent chemotherapy	Radiotherapy	OS	PFS	DMFS	LRFS
	Exp vs con	Exp vs con		Exp/con	Exp/con	Exp/con	Exp/con
Lv et al	Lob 30 mg/m^2^ d1,22 + 5FU 800 mg/m^2^d1–5,every 21 days for 2cycles vs DDP 100 mg/m^2^ + 5FU 800 mg/m^2^d1–5,every 21 days for 2cycles	Lob 30 mg/m^2^ 2 cycles vs DDP 100 mg/m^2^ 2 cycles	IMRT	5 year: 88.2%/89%	75%/75.5%	86.6%/85%	87.7%/88.8%
Tang et al	Doc 65 mg/m2 d1 + Ned 80 mg/m2 d1, every 21 days for 2cycles vs Doc 65 mg/m2 d1 + DDP 80 mg/m2 d1, every 21 days for 2cycles	Ned 40 mg/m2 every week for 3cycles vs DDP 40 mg /m2 every week for 3cycles	IMRT	3 year: 87.5%/85.9%	3 year: 77.5%/74.9%	3 year: 86.7%/85.1%	3 year: 91.9%/91.7%
Linquan et al	NR	Ned 100 mg/m^2^d1 every 21 days for 3cycles vs DDP 100 mg/m^2^ every 21 days for 3cycles	IMRT	5 year: 88.8%/89.4%	2 year: 88.0%/89.9% 5 year: 79.8%/81.4%	5 year: 90.4%/85.9%	5 year: 89.6%/92.6%
Liu et al	DDP 75 mg/m2 d1 + 5FU 800 mg/m^2^d1–5, every 21 days for 2–3 cycles vs Ned 75 mg/m^2^ + 5FU 800 mg/m^2^d1–5, every 21 days for 2–3 cycles	Ned 75 mg/m^2^d1 every 21 days for 2cycles vs DDP 80 mg/m^2^d1 every 21 days for 2cycles	IMRT	3 year: 82.4%/79.4%	3 year: 72.6%/68.7%	3 year: 80.7%/77.0%	3 year: 86.2%/92.6%
Zhan et al	Doc 60–75 mg/m2 d1 + DDP 60–75 mg/m2 d1 + 5FU 500–600 mg/m2, d1–5, every 21 days for 1–4 cycles	Ned 80 mg/m2 d1, every 21 days orNed 30 mg/m2 d1 every week vs DDP 80 mg/m2 d1,every 21 days or DDP 30 mg/m2 d1 every week	IMRT	3 year: 90.7%/92.3%	3 year: 78.9%/79.4%	3 year: 82.4%/85.1%	3 year: 96.1%/93.3%
Imjai et al	NR	CBP 100 mg/m2 every week vs DDP 100 mg/m^2^d1 every 21 days for 3cycles	2D-CRT	3 year: 77.7%/79.2%	NR	3 year: 63.4%/60.9%	NR

*5FU* 5-fluorouracil, *Lob* lobaplatin, *Doc* Docetaxel, *Ned*, nedaplatin, *DDP* cisplatin, *CBP* carboplatin, *OS* overall survival, *PFS* Progressive-free survival, *DMFS* distant metastasis-free survival, *LRFS* locoregional relapse-free survival, *FE* fail to extract

### OS

**Fig. 3:**
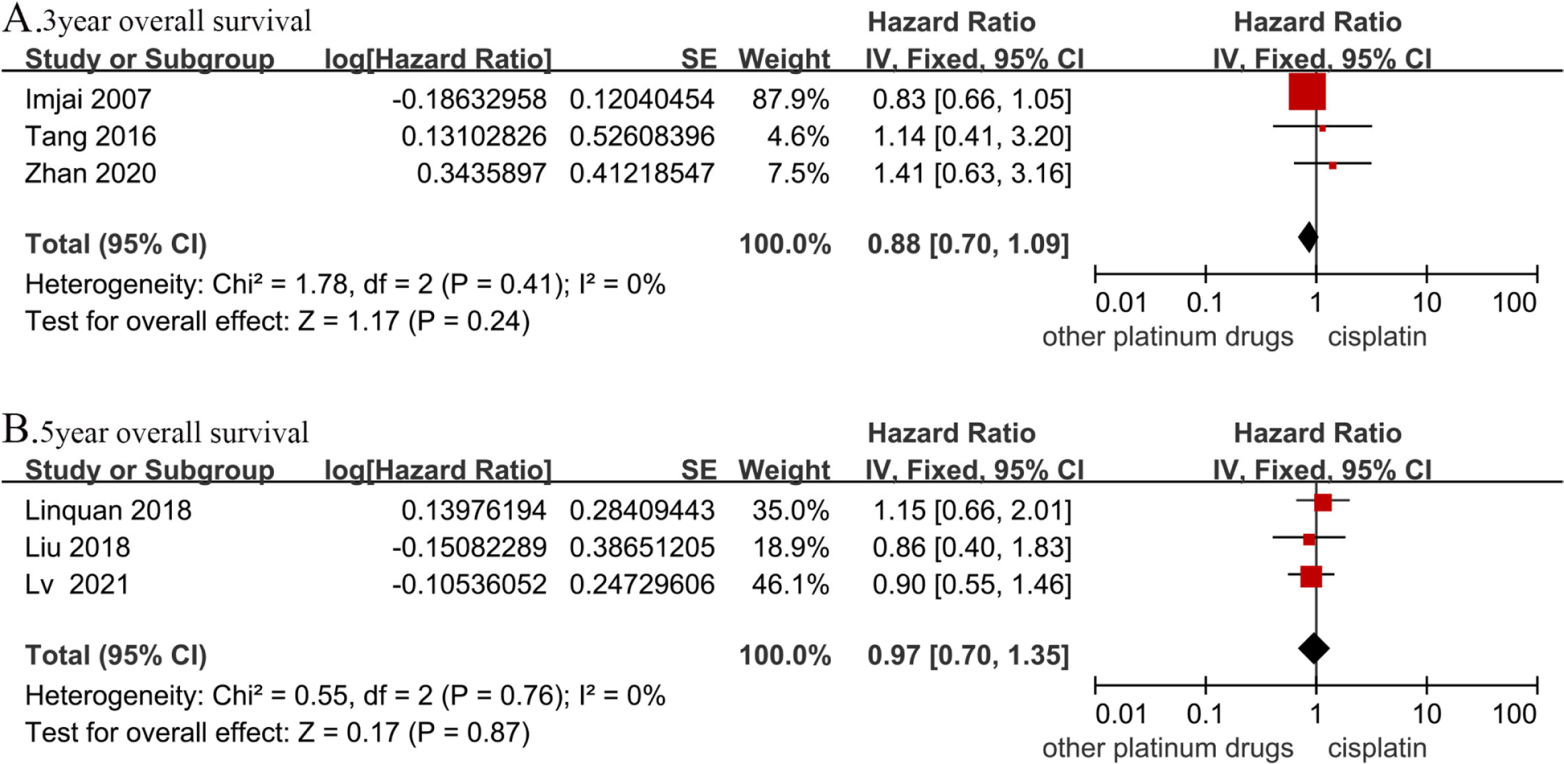
Forest plots of hazard ratios for (**A**) 3-year and (**B**) 5-year overall survival in nasopharyngeal carcinoma

The 3-year OS data were obtained from three studies with a total of 655 patients (cisplatin group, 328 patients; and other platinum-based chemotherapies group, 327 patients). Forest plots showed that there was no significant difference in the 3-year OS between the two groups (HR, 0.88; 95% CI, [0.70–1.09]; *p* = 0.24; H: I^2^ = 0%, *p* = 0.41). The 5-year OS data were obtained from three studies with a total of 1,090 patients (cisplatin group, 534 patients; and other platinum-based chemotherapies group, 556 patients). There was no significant difference in the 5-year OS between the two groups (HR, 0.97; 95% CI, [0.70–1.35]; *p* = 0.87; H: I^2^ = 0%; *p* = 0.76; Fig. 3).

### PFS

**Fig. 4:**
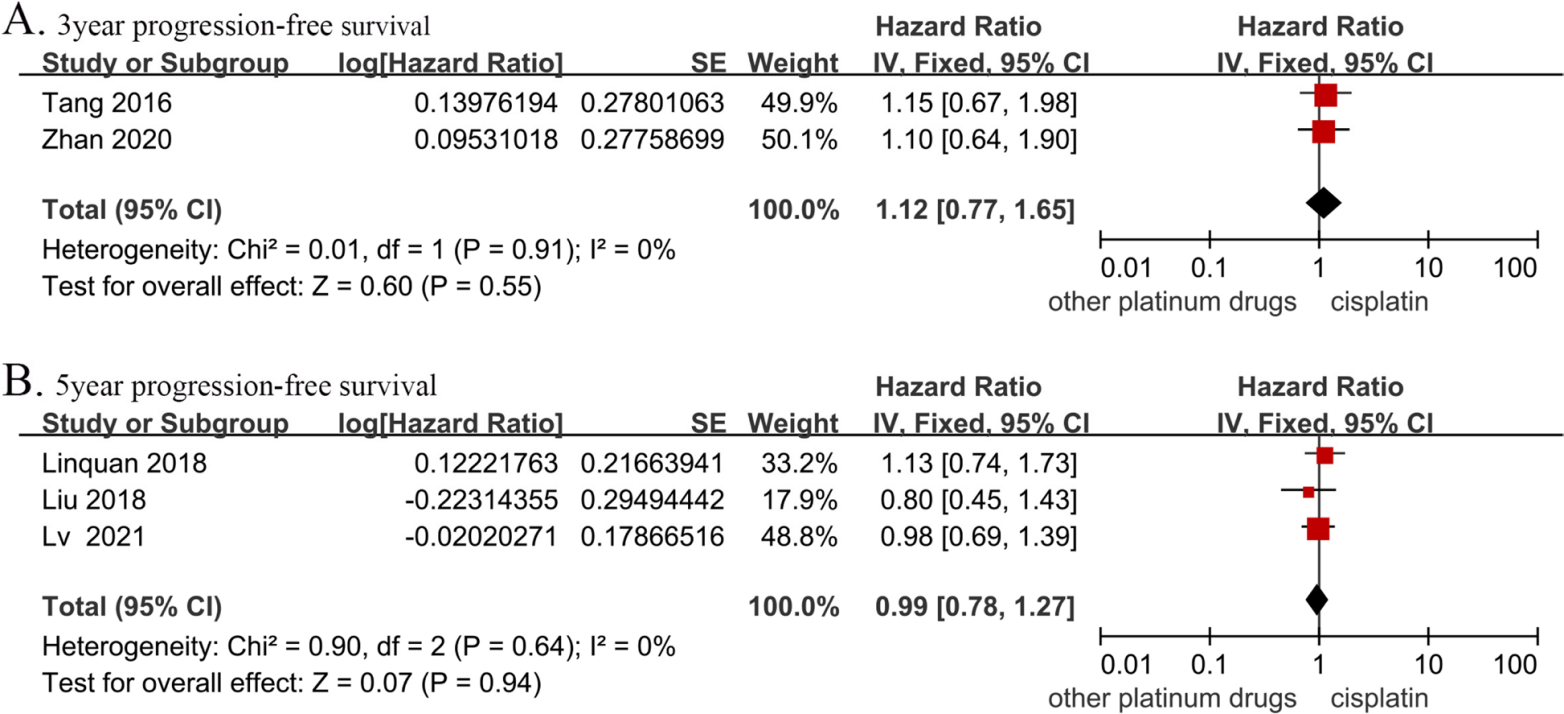
Forest plots of hazard ratios for (**A**) 3-year and (**B**) 5-year progression-free survival in nasopharyngeal carcinoma

The 3-year PFS data were obtained from 449 patients in two studies (cisplatin group, 223 patients; and other platinum-based chemotherapies group: 226 patients). There was no significant difference in the 3-year PFS between the two groups (HR, 1.12; 95% CI, [0.77–1.65]; *p* = 0.55; H: I^2^ = 0%; *p* = 0.91). The 5-year PFS data were obtained from three studies with a total of 1,090 patients (cisplatin group, 534 patients; and other platinum-based chemotherapies group, 556 patients). There was no significant difference in the 5-year PFS between the two groups (HR, 0.99; 95% CI, [0.78–1.27]; *p* = 0.94; H: I^2^ = 0%; *p* = 0.64) (Fig. 4).

### DMFS

**Fig. 5:**
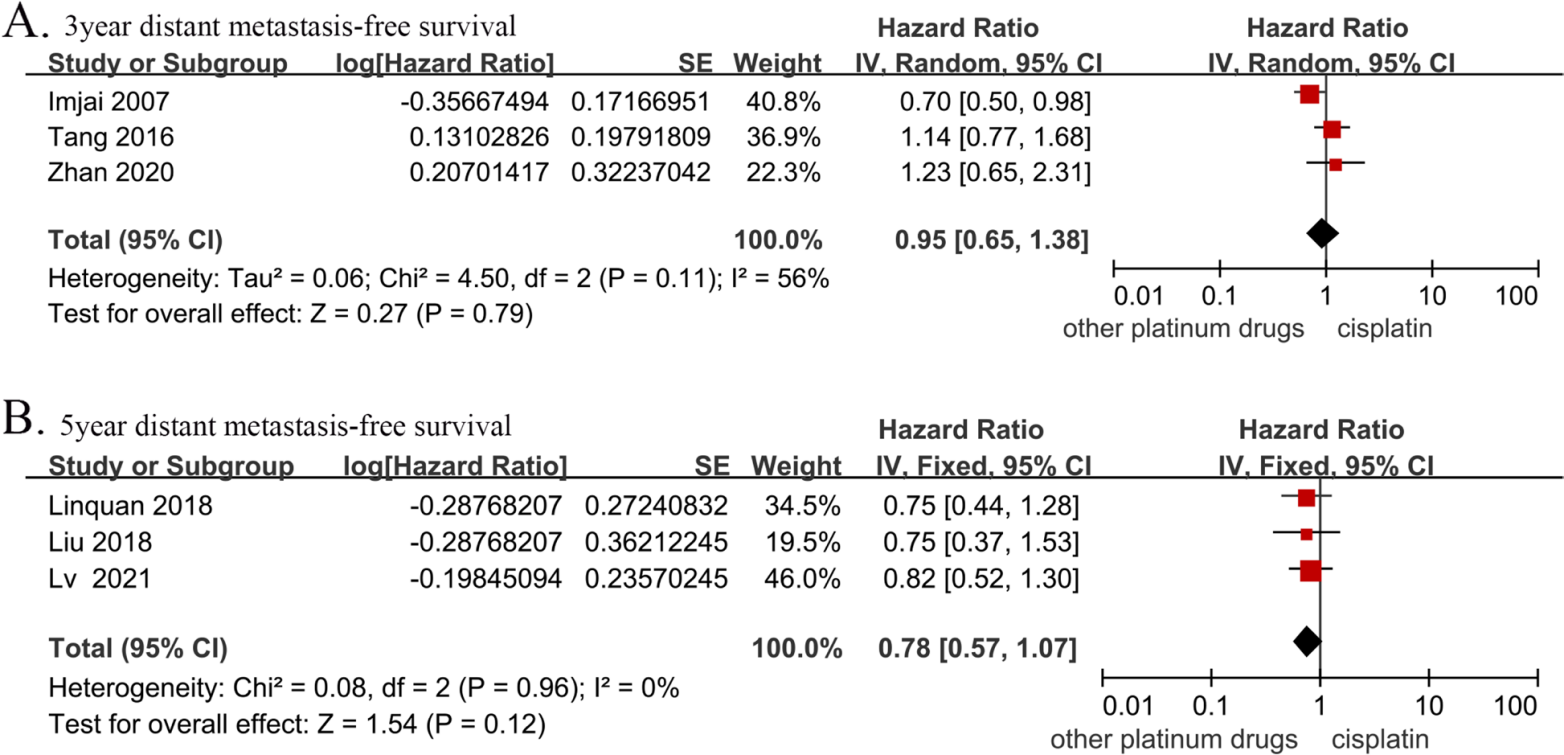
Forest plots of hazard ratios for (**A**) 3-year and (**B**) 5-year distant metastasis-free survival in nasopharyngeal carcinoma.

The 3-year DMFS data were obtained from a total of 655 patients in three studies (cisplatin group, 328 patients; and other platinum-based chemotherapies group, 327 patients). There was no significant difference in the 3-year DMFS between the two groups (HR, 0.95; 95% CI, [0.65–1.38]; *p* = 0.79; H: I^2^ = 56%; *p* = 0.11). The 5-year DMFS data were obtained from 1,090 patients in three studies (cisplatin group, 534 patients; and other platinum-based chemotherapies group, 556 patients). There was no significant difference in the 5-year DMFS between the two groups (HR, 0.78; 95% CI, [0.57–1.07]; *p* = 0.12; H: I^2^ = 0%; *p* = 0.96) (Fig. 5).

### LRFS

**Fig. 6:**
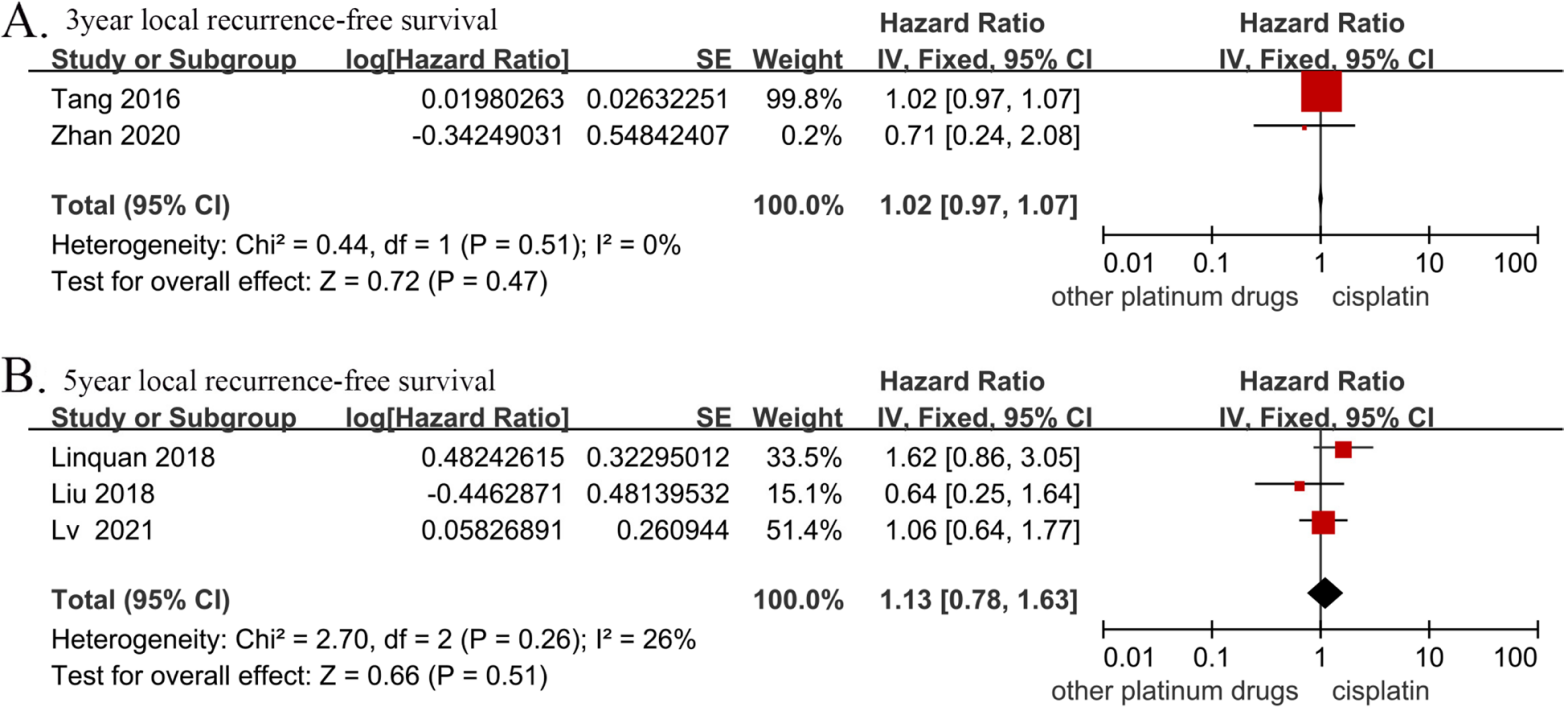
Forest plots of hazard ratios for (**A**) 3-year and (**B**) 5-year local recurrence-free survival in nasopharyngeal carcinoma

The 3-year LRFS data were obtained from a total of 449 patients in two studies (cisplatin group, 223 patients; and other platinum-based chemotherapies group, 226 patients). There was no significant difference in the 3-year LRFS between the two groups (HR, 1.02; 95% CI, [0.97–1.07]; *p* = 0.47; H: I^2^ = 0%; *p* = 0.51). The 5-year LRFS data were obtained from three studies with a total of 1,090 patients (cisplatin group, 534 patients; and other platinum-based chemotherapies group, 556 patients). There was no significant difference in the 5-year LRFS between the two groups (HR, 1.13; 95% CI, [0.78–1.63]; *p* = 0.51; H: I^2^ = 26%; *p* = 0.26) (Fig. 6).

### Grade ≥ 3 acute toxicities

Based on acute grade 3 or higher acute toxicities during treatment in the other platinum-based chemotherapies and cisplatin groups, the following risks were calculated. With regard to hematological toxicities, there was no significant difference in the risk of neutropenia (RR, 1.21; 95% CI, [0.94–1.57]; *p* = 0.14), leukopenia (RR, 0.97; 95% CI, [0.81–1.17]; *p* = 0.78), or thrombocytopenia (RR, 1.62; 95% CI, [0.98–2.69]; *p* = 0.06) between the other platinum-based chemotherapies group and the cisplatin group. However, the risk of anemia in the other platinum-based chemotherapies group was significantly higher than that of the cisplatin group (RR, 0.30; 95% CI, [0.12–0.77]; *p* = 0.01).

With regard to non-hematological toxicities, there was no significant difference in the risk of xerostomia (RR, 0.83; 95% CI, [0.51–1.35]; *p* = 0.46), dermatitis (RR, 1.02; 95% CI, [0.58–1.81]; *p* = 0.95), mucositis (RR, 1.02; 95% CI, [0.58–1.81]; *p* = 0.95), or elevated levels of aminotransferase (RR, 0.71; 95% CI, [0.25–2.05], *p* = 0.53) between the other platinum-based chemotherapies group and the cisplatin group. However, the risk of nausea (RR, 0.12; 95% CI, [0.06–0.25]; *p* < 0.0001), vomiting (RR, 0.15; 95% CI, [0.06–0.40]; *p* = 0.0001), and weight loss (RR, 0.34; 95% CI, [0.12–0.98], *p* = 0.04) were significantly lower in the other platinum-based chemotherapies group than those in the cisplatin group (Table 3).

**Table 3:** Grade 3–4 acute toxicities during treatment

Advese event (grade3-4)	Availability			Effect		Heterogeneity		Analysis model
	Trials (N)	Other platinum (events/total)	Cisplatin (events/total)	RR (95% CI)	*P* value	I^2^	*P* value	
Hematological								
neutropenia	5	210/781	169/753	1.21(0.94–1.57)	0.14	53%	0.07	Random effect
leucopenia	5	177/773	173/744	0.97(0.81–1.17)	0.78	45%	0.12	Fixed effect
thrombocytopenia	6	88/886	56/854	1.62(0.98–2.69)	0.06	51%	0.07	Random effect
anemia	5	26/783	77/771	0.30(0.12–0.77)	0.01	72%	0.007	Random effect
Nonhematologic								
xerostomia	5	28/773	33/741	0.83(0.51–1.35)	0.46	0%	0.48	Fixed effect
dermatitis	4	24/573	22/543	1.02(0.58–1.81)	0.95	0%	0.4	Fixed effect
mucositis	6	211/886	227/854	0.91(0.78–1.06)	0.23	27%	0.24	Fixed effect
nausea	3	8/555	62/530	0.12(0.06–0.25)	< 0.0001	0%	0.41	Fixed effect
vomiting	5	22/781	125/753	0.15(0.06–0.40)	0.0001	61%	0.04	Random effect
weight loss	3	4/468	12/445	0.34(0.12–0.98)	0.05	30%	0.24	Fixed effect
Elevation of aminotransferase	2	6/135	7/114	0.71(0.25–2.05)	0.53	0%	0.81	Fixed effect

### Treatment-related late toxicities

**Fig. 7:**
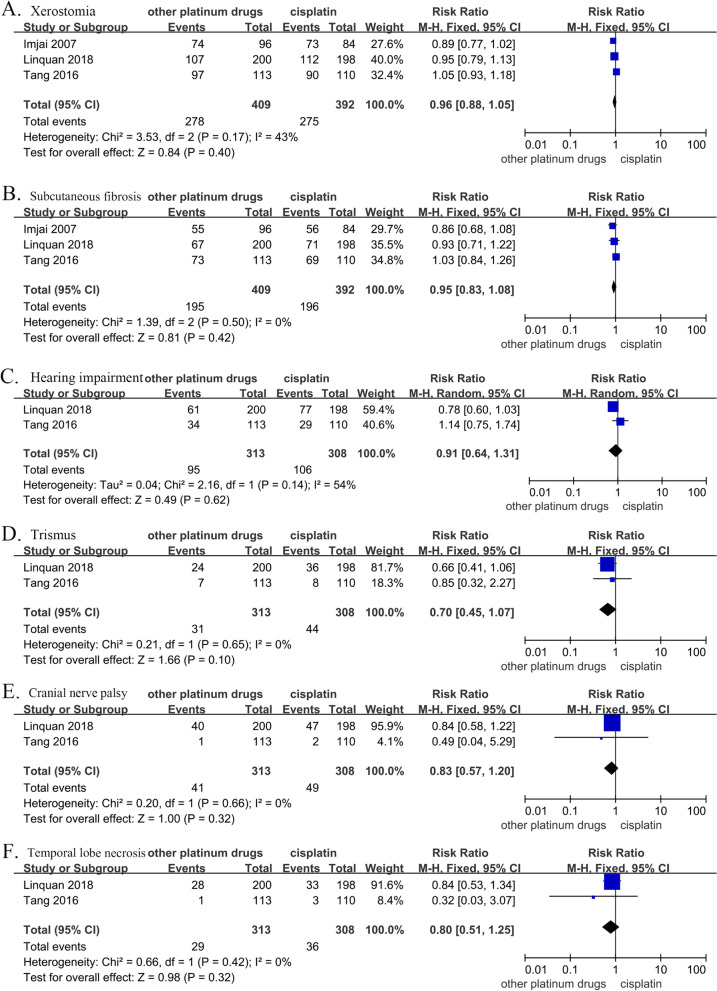
Forest plots of risk ratios for cumulative grade 1–2 late toxicities, including: (**A**) xerostomia, (**B**) subcutaneous fibrosis, (**C**) hearing impairment, (**D**) trismus, (**E**) cranial nerve palsy, (**F**) temporal lobe necrosis

Based on the late adverse events during the treatment with other platinum derivatives and cisplatin, there was no significant difference between the two groups regarding the risk of xerostomia (RR, 0.96; 95% CI, [0.88–1.05]; *p* = 0.40), subcutaneous fibrosis (RR, 0.95; 95% CI, [0.83–1.08]; *p* = 0.42), hearing impairment (RR, 0.91; 95% CI, [0.64–1.31]; *p* = 0.62), trismus (RR, 0.70; 95% CI, [0.45–1.07]; *p* = 0.10), cranial nerve palsy (RR, 0.83; 95% CI, [0.57–1.20], *p* = 0.32), or temporal lobe necrosis (RR, 0.80; 95% CI, [0.51–1.25]; *p* = 0.32) (Fig. 7).

### Subgroup and sensitivity analyses

**Fig. 8:**
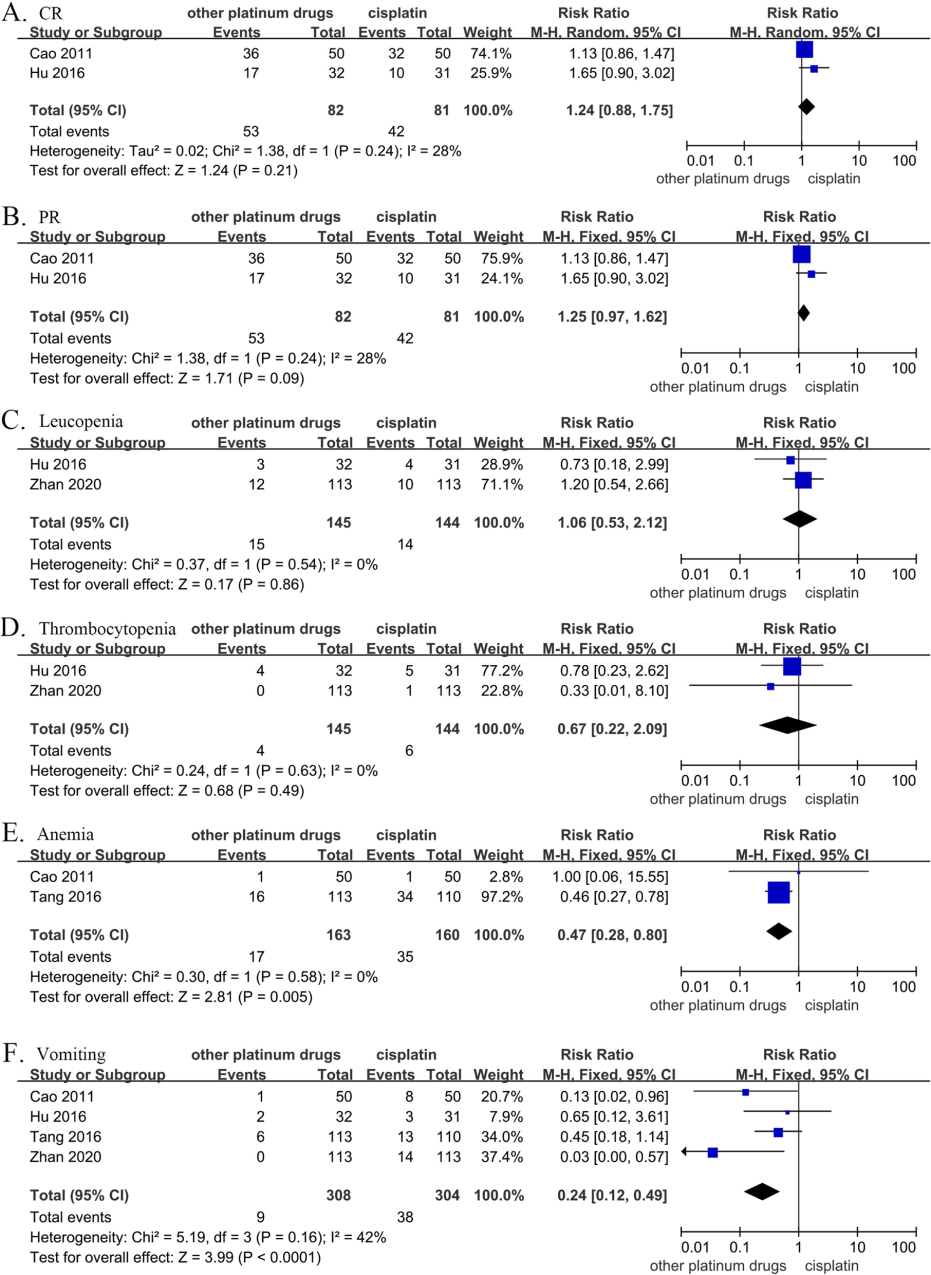
Forest plots of risk ratios for the cumulative response rates and toxicities of induced chemotherapy

Two studies reported the efficacy and side effects of induction chemotherapy alone [18, 21], so these two outcomes were analyzed separately. After induction chemotherapy, there was no significant difference in complete response (RR, 1.24; 95% CI, [0.88–1.75.08]; *p* = 0.21) or partial response (RR, 1.25; 95% CI, [0.97–1.62]; *p* = 0.09) between the other platinum-based chemotherapies group and the cisplatin group. There was also no significant difference in the risk of leukocytopenia (RR, 1.06; 95% CI, [0.53–2.12]; *p* = 0.86) or thrombocytopenia (RR, 0.67; 95% CI, [0.22–2.09]; *p* = 0.49) between the two groups. However, the risk of anemia (RR, 0.47; 95% CI, [0.28–0.80]; *p* = 0.005) was significantly higher in the other platinum-based chemotherapies group than that of the cisplatin group. Moreover, the incidence of vomiting (RR, 0.24; 95% CI, [0.12–0.49]; *p* < 0.0001) in the cisplatin group was significantly higher than that of the other platinum-based chemotherapies group. The sensitivity analysis showed that the aggregated results at all endpoints remained unchanged when any study was deleted, indicating that the results of this meta-analysis are reliable (Fig. 8).

## Discussion

The study showed that the other platinum-based chemotherapy alternatives did not reduce survival and did not significantly increase the incidence of hematological and non-hematological side effects compared with cisplatin-based chemotherapy. To the best of our knowledge, this is the first meta-analysis to examine the efficacy and side effects of cisplatin versus other platinum-based chemotherapies in locally advanced NPC.

In the past 20 years, three major advances have significantly improved the prognosis of patients with NPC. First, intensity-modulated radiation therapy can cover the target area and the local expansion area with good precision. Intensity-modulated radiation therapy can better protect the adjacent normal tissue, especially for patients whose tumors extend backward to the cranial nerve [23, 24]. Second, the combination of cisplatin-based CCRT, induction chemotherapy, or adjuvant chemotherapy effectively improves the survival rate and disease control of NPC [3, 5, 25–27]. Third, the use of advanced imaging techniques, especially the application of MRI and PET-CT, can better evaluate the local and distant invasion of the tumor, which is very critical for the accurate application of intensity-modulated radiation therapy. However, cisplatin-based chemotherapy regimens are known to increase the acute and late toxicities of radiotherapy [16]. Long-term side effects such as nausea, vomiting, auditory function, renal function, or effects on peripheral nerves caused by cisplatin may affect the quality of life of survivors. Moreover, cisplatin-based CCRT requires pretreatment and post-treatment hydration during cisplatin administration to protect the kidneys, which can prolong the hospital stay [14, 16, 17].

Carboplatin, nedaplatin, and lobaplatin were successively included in the study as cisplatin substitutes to improve the compliance of patients, reduce the side effects of chemotherapy and meet the clinical needs. A randomized non-inferiority trial showed that there was no difference between carboplatin-based CCRT and a cisplatin-based regimen in patients with locally advanced NPC. Moreover, carboplatin showed better tolerance in patients with locally advanced NPC [22]. Two other trials indicated that carboplatin induction chemotherapy combined with CCRT did not improve survival in patients with locally advanced NPC compared with carboplatin induction chemotherapy combined with radiotherapy alone [9]. In addition, carboplatin was less effective than cisplatin when given during CCRT in patients with borderline renal function [28].

Nedaplatin, a cisplatin analog, has antitumor mechanism and therapeutic effects similar to that of cisplatin and does not require hydration to protect the kidneys. Two Phase 2 studies have shown that nedaplatin in combination with fluorouracil or docetaxel has an inductive effect on chemotherapy. In addition, nedaplatin-based CCRT is an effective and safe treatment for patients with stage II–IVB NPC, indicating that nedaplatin may be a promising alternative to cisplatin [15, 29]. In a randomized phase III trial, Mai et al*.* [16] showed that for patients with stages II–IVB NPC, nedaplatin-based CCRT was not inferior to cisplatin-based CCRT with respect to the 2-year PFS. Subsequent comments [30] indicate that it is too early to conclude that nedaplatin will replace cisplatin. However, the newly published results of the 5-year follow-up still support the results of the initial report [17].

Lobaplatin is a third-generation platinum drug. In previous studies, lobaplatin was found to overcome some forms of multiple drug resistance caused by other platinum-based drugs, such as cisplatin or carboplatin [8]. A random non-inferiority trial showed that lobaplatin-based induction chemotherapy plus CCRT has similar survival outcomes and side effect profiles as cisplatin-based therapy and thus may act as a promising alternative [14]. Clinical studies, such as ChiCTR1900021536 and ChiCTR-IIR-17013112, are ongoing and aim to further assess the benefits and risks of lobaplatin for NPC and verify the value of these treatment strategies.

Cisplatin has a lower drug price than platinum derivatives; however, it also has more symptomatic adverse events that require additional treatment processes, such as hydration and antiemetic preconditioning, and this increases the cost of treatment accordingly [31]. Liao et al*.* [10] found that nedaplatin is an advantageous and low-cost alternative to concurrent chemoradiotherapy for stage II–IVB NPC, based on a cost–benefit curve analysis. Lv et al*.* [14] mentioned that in south China, an area with high incidence of NPC, although the price of a new generation of platinum derivatives is higher than that of cisplatin, various chemotherapy drugs (such as lobaplatin) are included in the list of essential drugs under China's medical insurance system, and the supply of generic drugs reduces the cost [32]. However, the limited number of inpatient beds and the length of stay pose challenges. Patients waiting for hospital treatment may experience disease progression and have increased psychological stress. Shorter hospital stays with cisplatin derivatives may help alleviate these problems.

We conducted this meta-analysis to evaluate the efficacy and safety of other platinum-based chemotherapies versus cisplatin-based chemotherapy for locally advanced NPC. Choi et al. [33] performed a network meta-analysis on the efficacy of different neoadjuvant chemotherapeutic strategies in the treatment of NPC. The results showed that some cisplatin-based neoadjuvant chemotherapy regimens improved the prognosis of patients with NPC and reduced the toxicity of chemotherapy. However, the optimal neoadjuvant chemotherapy protocol is not fully consistent in terms of survival and efficiency. Yuan et al*.* [34] showed that the induction chemotherapy regimen, gemcitabine plus cisplatin, shows better performance in terms of survival outcomes. To date, there is no meta-analysis to adequately demonstrate differences in the efficacy of various platinum-based regimens in locally advanced NPC. To reduce bias, we selected RCTs that are clinically registered as eligible studies. Our meta-analysis revealed that there was no significant difference between other platinum-based and cisplatin-based chemotherapy in terms of OS, PFS, DMFS, and LRFS. Severe acute hematological side effects (≥ grade 3) such as neutropenia, leukopenia and thrombocytopenia were observed after platinum-based induction chemotherapy or throughout the treatment period; however, such side effects were equivalent to those in the cisplatin treatment group. It is worth noting that the risk of anemia was higher in patients receiving other platinum-based treatments. In contrast, the risk of non-hematological side effects such as nausea, vomiting, and weight loss after induction chemotherapy or during the whole treatment period was higher in the cisplatin treatment group. There was no difference in other non-hematological side effects, such as xerostomia, dermatitis, mucositis, and elevated levels of aminotransferase, between the two groups. Moreover, there was no significant difference in the late side effects such as xerostomia, subcutaneous fibrosis, hearing impairment, trismus, cranial nerve palsy and temporal lobe necrosis between the two groups. The studies included in this meta-analysis did not report any treatment-related disability or death.

The main limitation of this meta-analysis is that some of the studies included were not RCTs, which may affect our research outcomes. Moreover, most studies were conducted in China, which may be a source of potential bias. In addition, there are differences in the specific study populations, combined treatment schemes and treatment durations, which may affect further data analyses. Finally, the DNA level of EB virus is a prognostic factor for NPC, however, the included studies could not be analyzed by subgroups to address this factor.

## Conclusion

Based on the systematic review and meta-analysis of the included studies, other platinum-based chemotherapy regimens were not inferior to cisplatin-based regimens and could be effective alternatives to cisplatin for the treatment of locally advanced NPC. Since most eligible studies were conducted in endemic areas, high-level evidence is needed to verify these findings in the future.

## Supplementary Information

**Additional file 1. **

## Data Availability

All data generated or analysed during this study are included in this published article.

## Abbreviations

NPC: Nasopharyngeal carcinoma
RCT: Randomized controlled trials
IC: Induction chemotherapy
CCRT: Concurrent chemoradiotherapy
AC: Adjuvant chemotherapy
Con: Control group (cisplatin-based group)
Exp: Experimental group (other platinum-based group)
NA: Not available
Re: Retrospetive study
NR: Not reported
FE: Fail to extract
5FU: 5-Fluorouracil
OS: Overall survival
PFS: Progressive-free survival
DMFS: Distant metastasis-free survival
LRFS: Locoregional relapse-free survival
