# Supplementary figures and images for: Comparing the efficacy and safety of cisplatin and other platinum-based chemotherapies in locally advanced nasopharyngeal carcinoma: a systematic review and meta-analysis
Source: BMC Cancer. 2022 Jun 6;22:616. doi: 10.1186/s12885-022-09712-z (PMC9169397; doi:10.1186/s12885-022-09712-z)

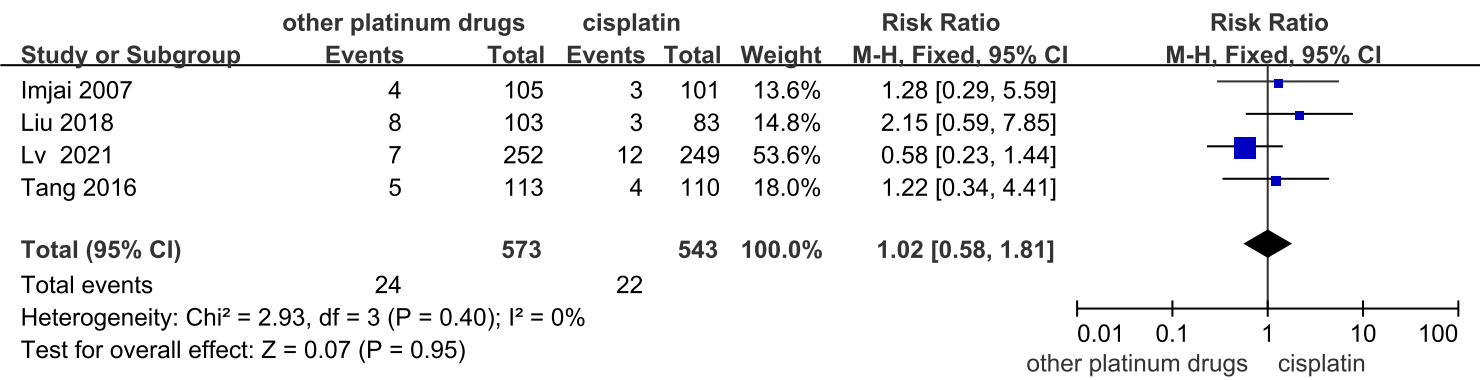

Supplement: Supplementary file 1 — Additional file 1. [file 12885_2022_9712_MOESM1_ESM.zip › Supplementary material/figure/3-4grade1/Dermatitis.pdf]

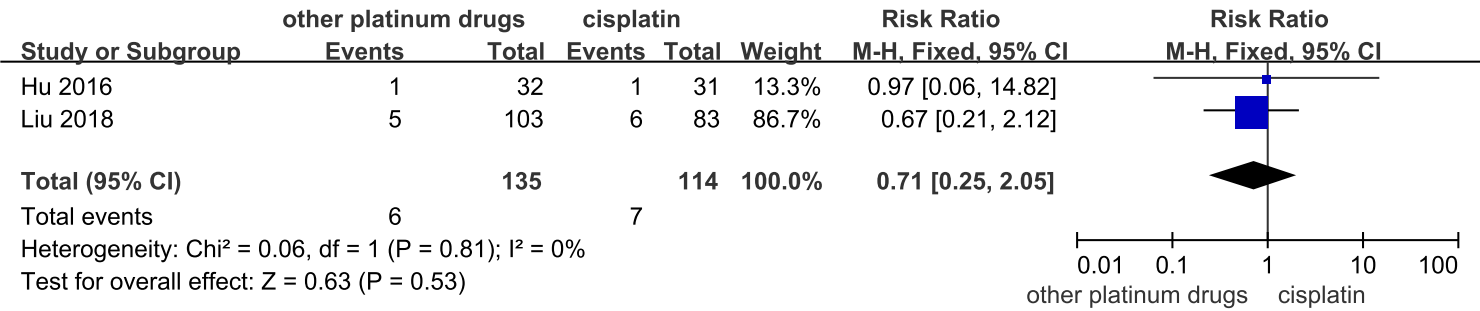

Supplement: Supplementary file 1 — Additional file 1. [file 12885_2022_9712_MOESM1_ESM.zip › Supplementary material/figure/3-4grade1/Elevation of aminotransferase.pdf]

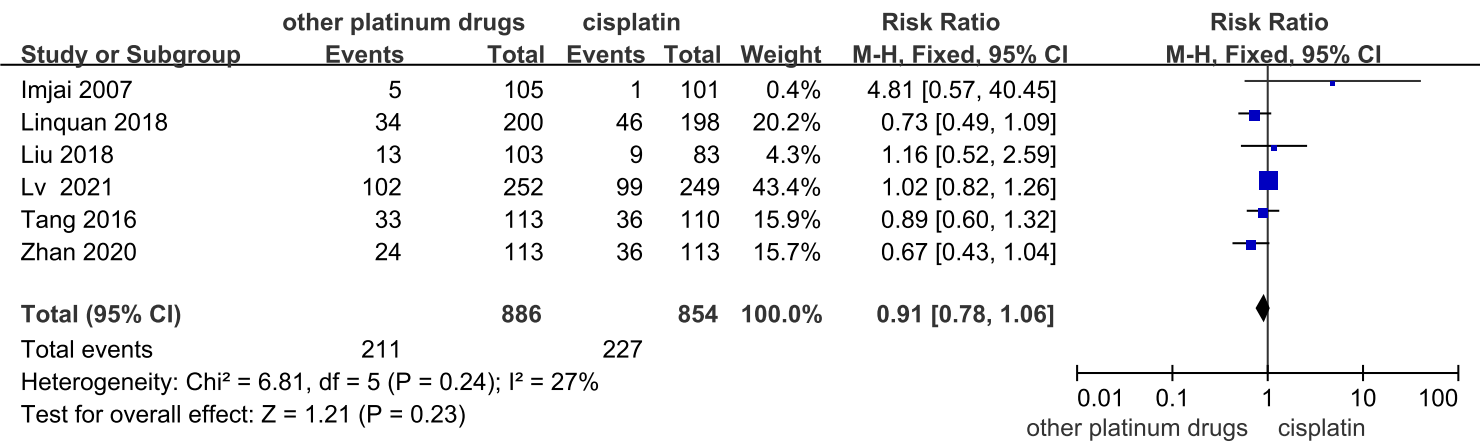

Supplement: Supplementary file 1 — Additional file 1. [file 12885_2022_9712_MOESM1_ESM.zip › Supplementary material/figure/3-4grade1/Mucositis.pdf]

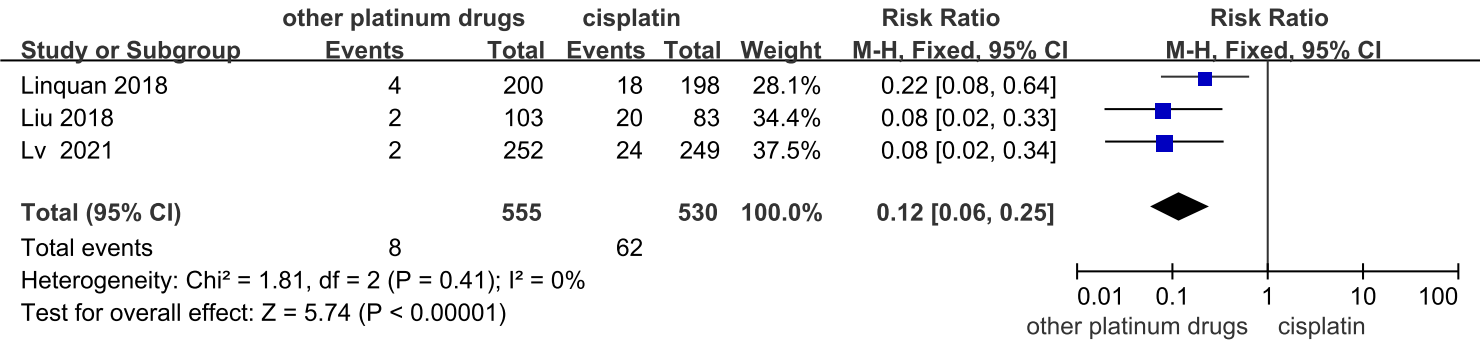

Supplement: Supplementary file 1 — Additional file 1. [file 12885_2022_9712_MOESM1_ESM.zip › Supplementary material/figure/3-4grade1/Nausea.pdf]

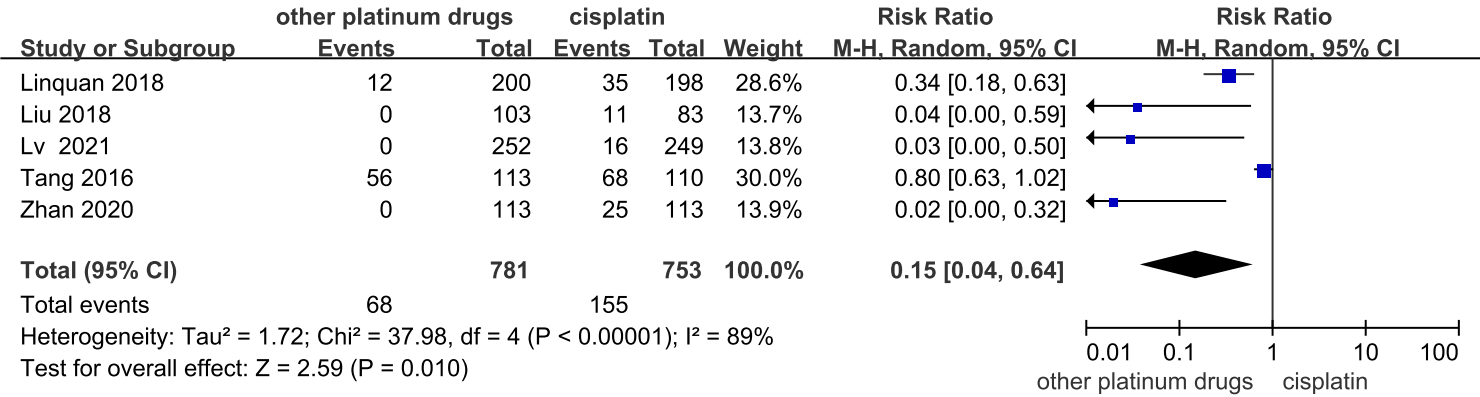

Supplement: Supplementary file 1 — Additional file 1. [file 12885_2022_9712_MOESM1_ESM.zip › Supplementary material/figure/3-4grade1/Vomiting.pdf]

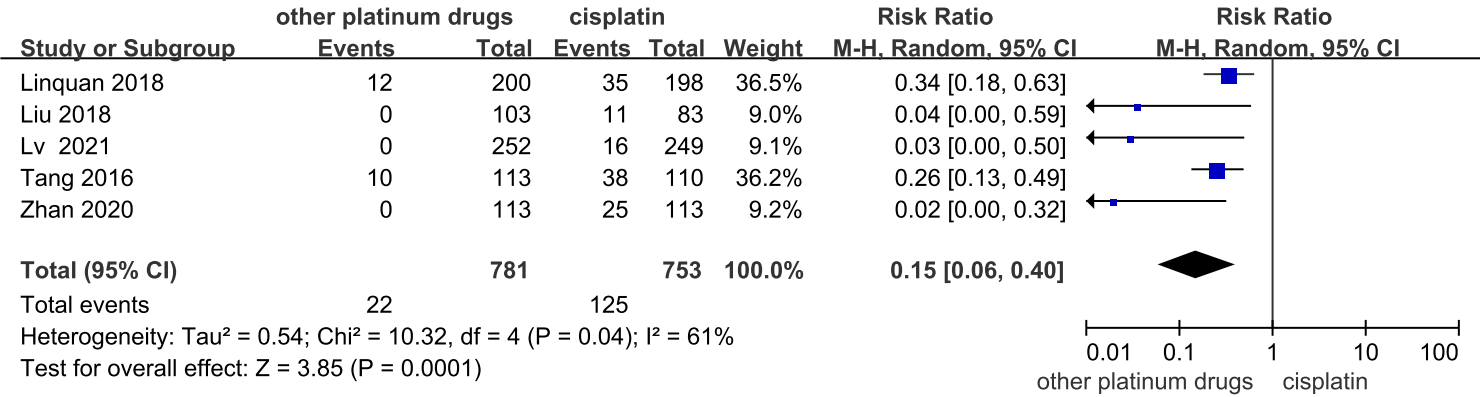

Supplement: Supplementary file 1 — Additional file 1. [file 12885_2022_9712_MOESM1_ESM.zip › Supplementary material/figure/3-4grade1/Vomiting1.pdf]

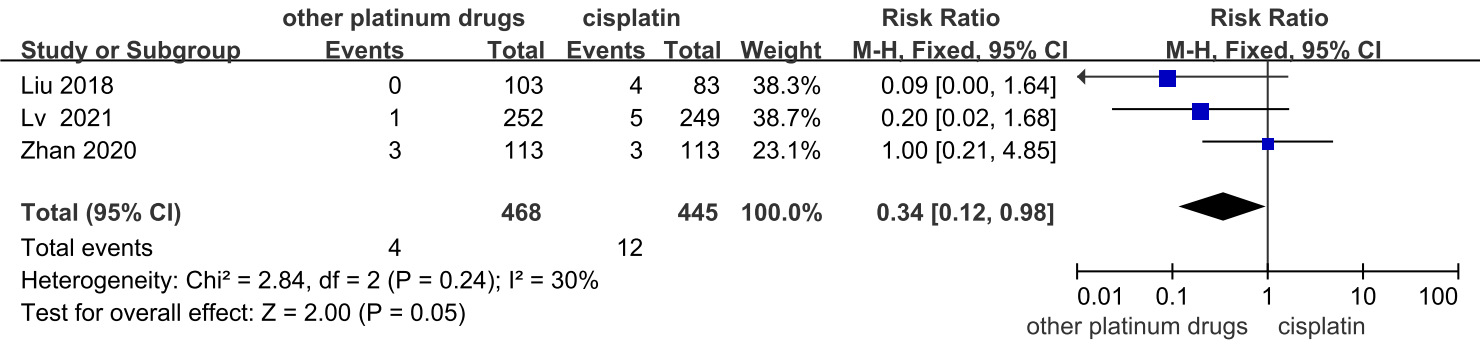

Supplement: Supplementary file 1 — Additional file 1. [file 12885_2022_9712_MOESM1_ESM.zip › Supplementary material/figure/3-4grade1/Weight loss.pdf]

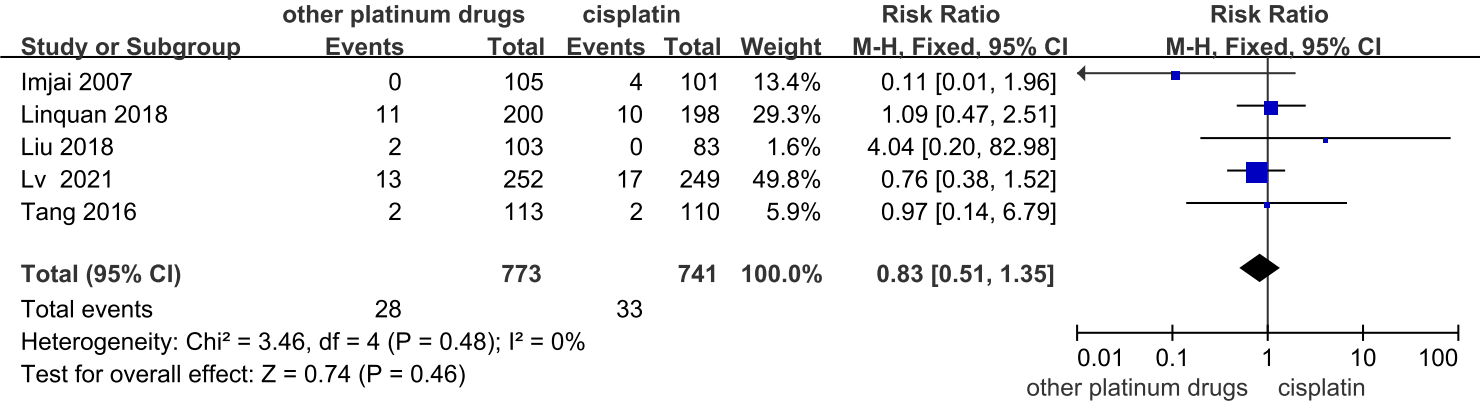

Supplement: Supplementary file 1 — Additional file 1. [file 12885_2022_9712_MOESM1_ESM.zip › Supplementary material/figure/3-4grade1/Xerostomia.pdf]

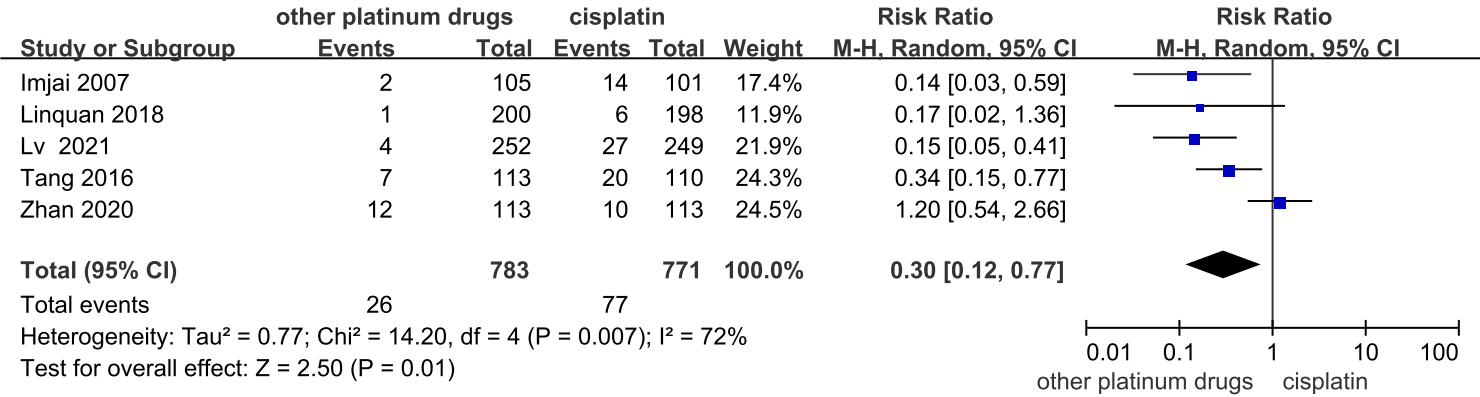

Supplement: Supplementary file 1 — Additional file 1. [file 12885_2022_9712_MOESM1_ESM.zip › Supplementary material/figure/3-4grade2/Anemia.pdf]

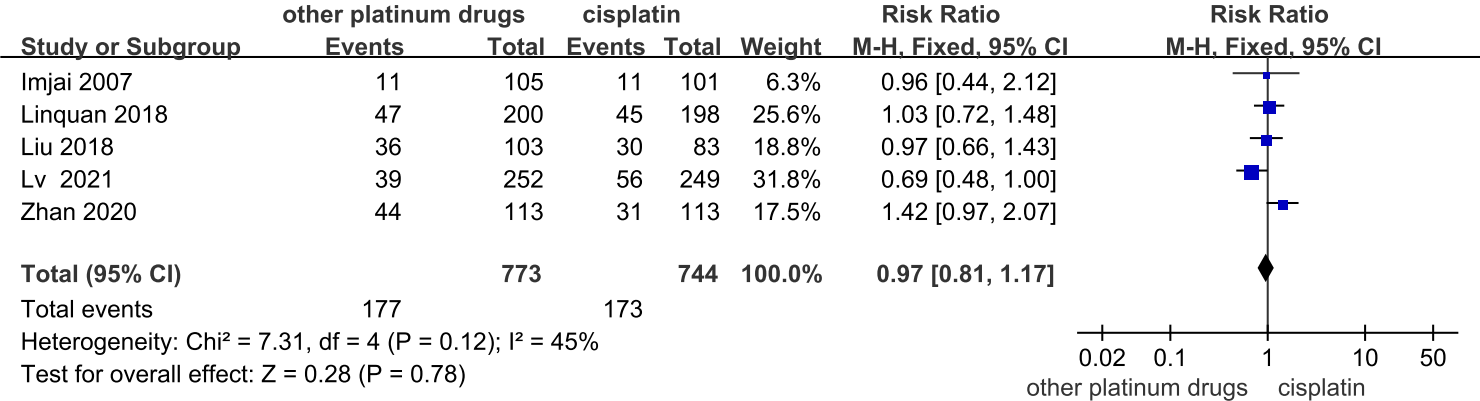

Supplement: Supplementary file 1 — Additional file 1. [file 12885_2022_9712_MOESM1_ESM.zip › Supplementary material/figure/3-4grade2/Leucopenia.pdf]

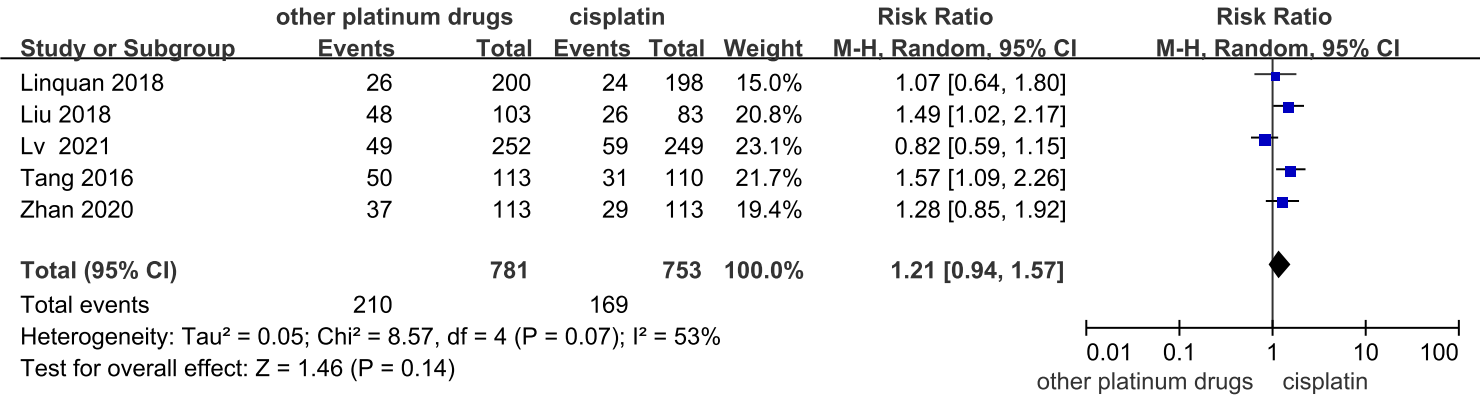

Supplement: Supplementary file 1 — Additional file 1. [file 12885_2022_9712_MOESM1_ESM.zip › Supplementary material/figure/3-4grade2/Neutropenia.pdf]

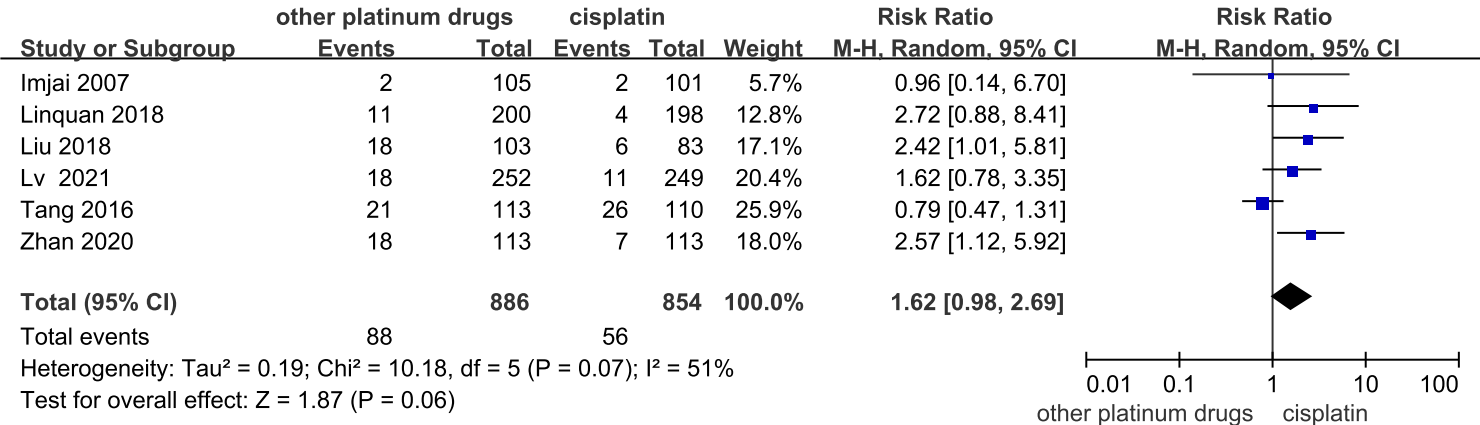

Supplement: Supplementary file 1 — Additional file 1. [file 12885_2022_9712_MOESM1_ESM.zip › Supplementary material/figure/3-4grade2/Thrombocytopenia.pdf]

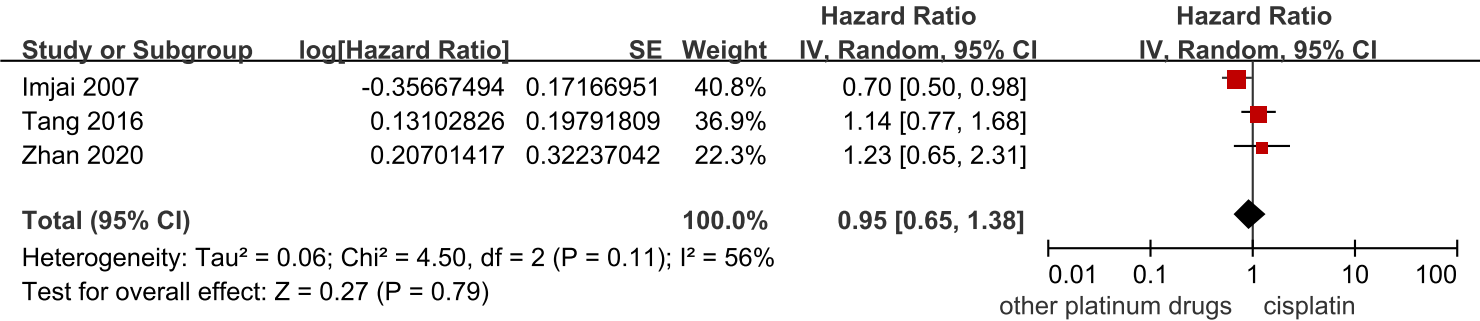

Supplement: Supplementary file 1 — Additional file 1. [file 12885_2022_9712_MOESM1_ESM.zip › Supplementary material/figure/DMFS/3DMFS.pdf]

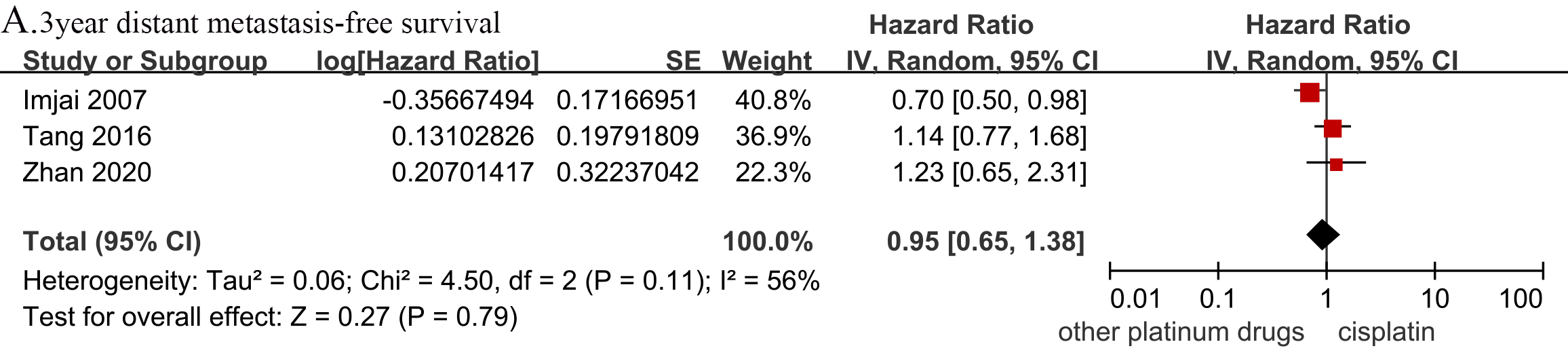

Supplement: Supplementary file 1 — Additional file 1. [file 12885_2022_9712_MOESM1_ESM.zip › Supplementary material/figure/DMFS/3DMFS.tif]

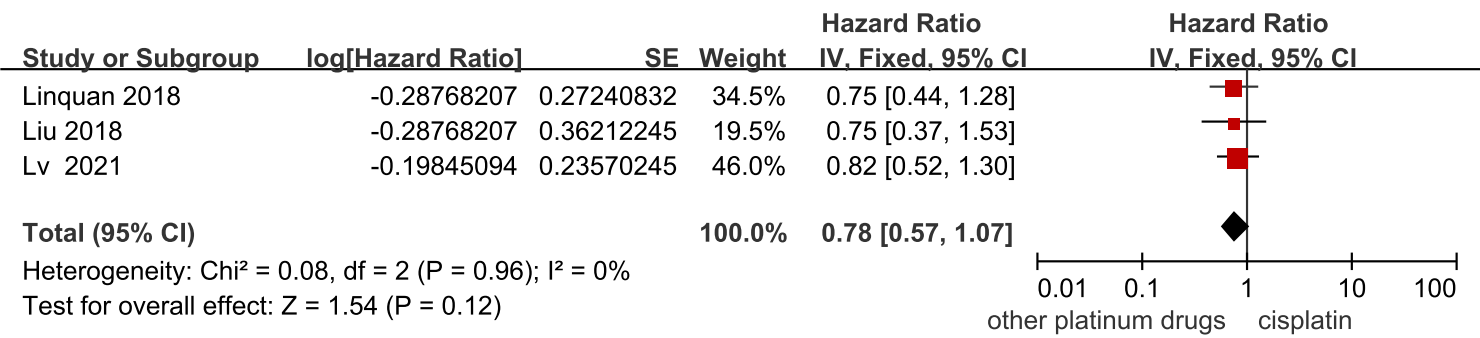

Supplement: Supplementary file 1 — Additional file 1. [file 12885_2022_9712_MOESM1_ESM.zip › Supplementary material/figure/DMFS/5DMFS.pdf]

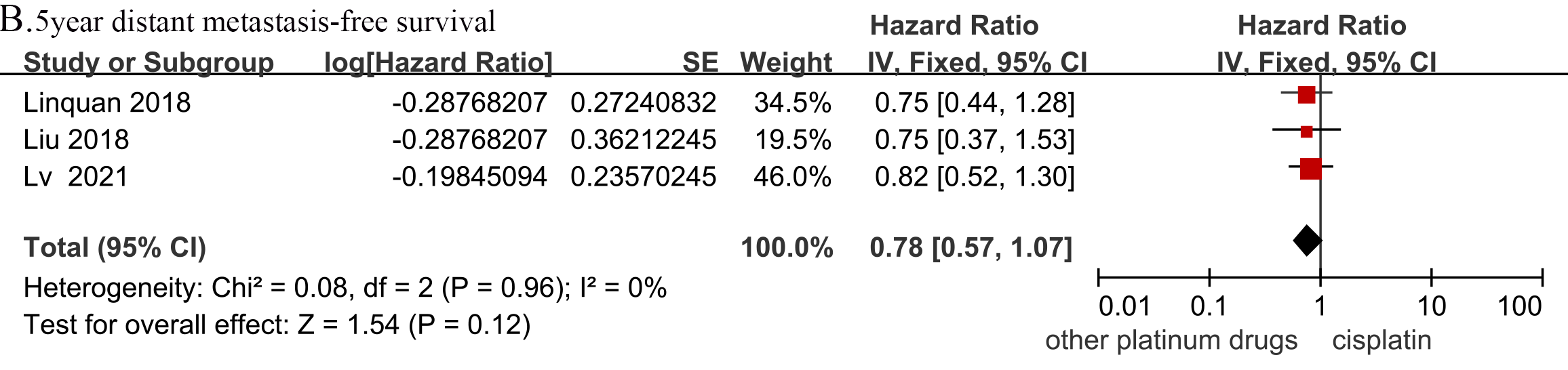

Supplement: Supplementary file 1 — Additional file 1. [file 12885_2022_9712_MOESM1_ESM.zip › Supplementary material/figure/DMFS/5DMFS.tif]

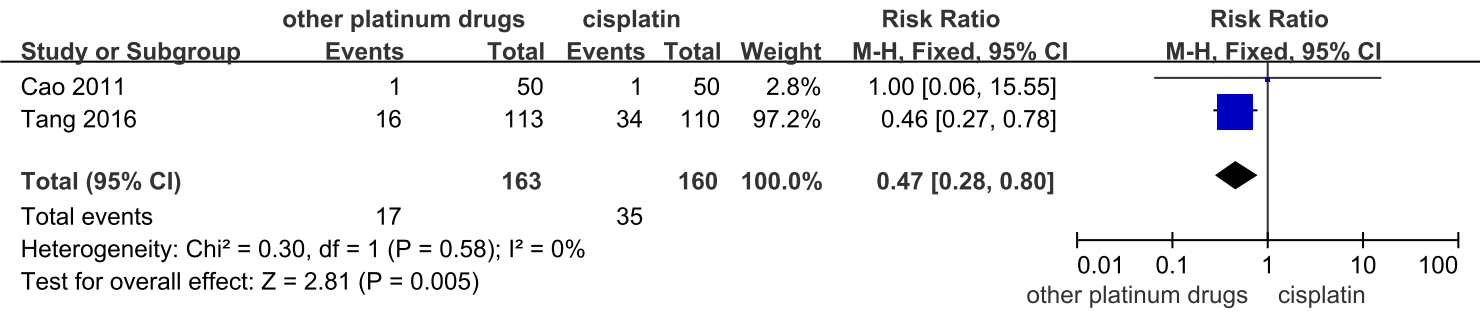

Supplement: Supplementary file 1 — Additional file 1. [file 12885_2022_9712_MOESM1_ESM.zip › Supplementary material/figure/Induction chemotherapy/Anemia .pdf]

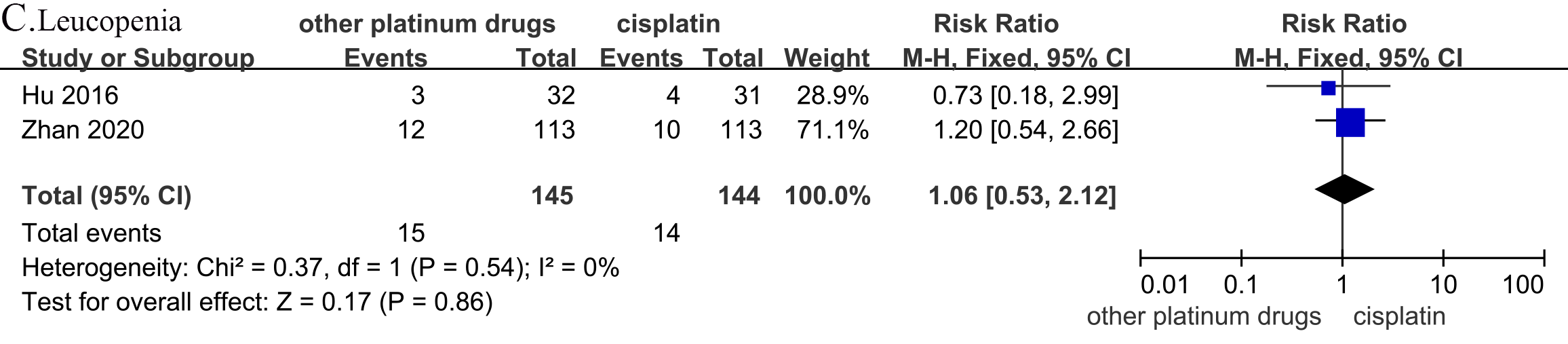

Supplement: Supplementary file 1 — Additional file 1. [file 12885_2022_9712_MOESM1_ESM.zip › Supplementary material/figure/Induction chemotherapy/C.tif]

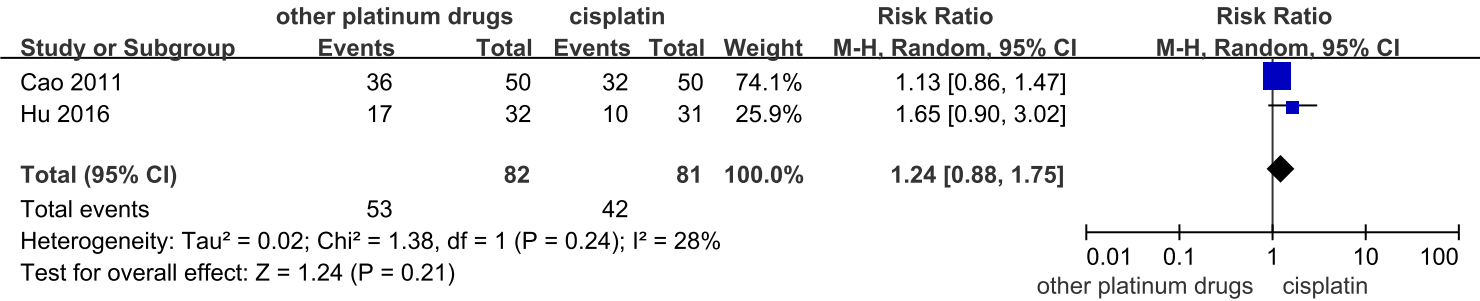

Supplement: Supplementary file 1 — Additional file 1. [file 12885_2022_9712_MOESM1_ESM.zip › Supplementary material/figure/Induction chemotherapy/CR.pdf]

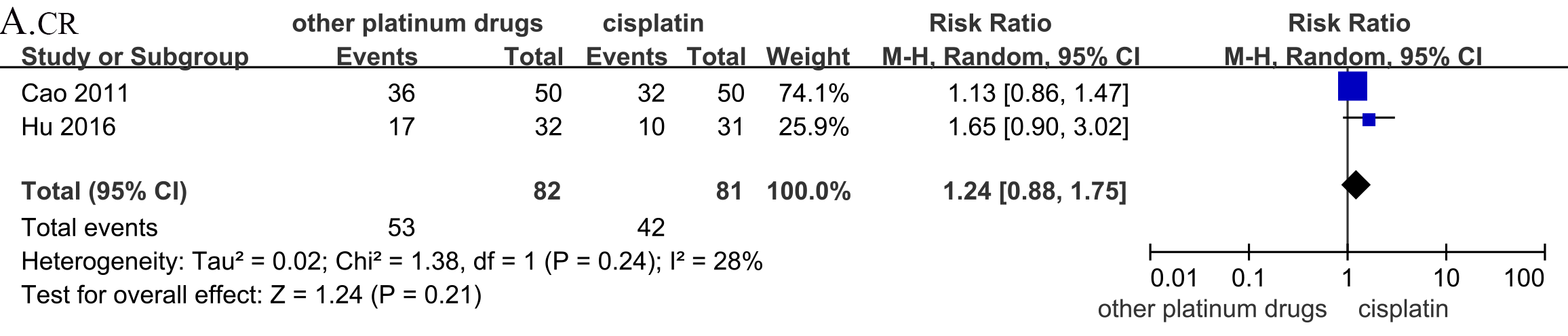

Supplement: Supplementary file 1 — Additional file 1. [file 12885_2022_9712_MOESM1_ESM.zip › Supplementary material/figure/Induction chemotherapy/CR.tif]

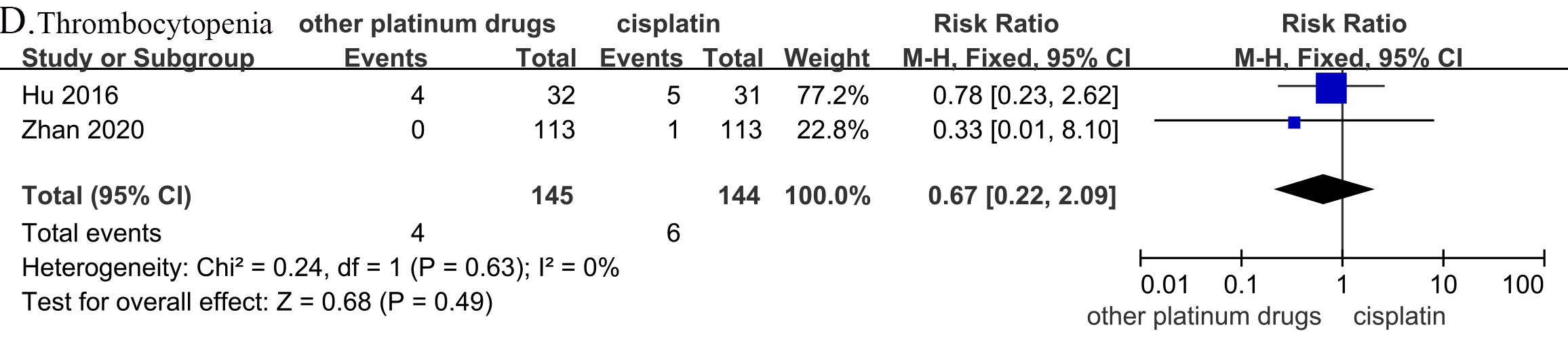

Supplement: Supplementary file 1 — Additional file 1. [file 12885_2022_9712_MOESM1_ESM.zip › Supplementary material/figure/Induction chemotherapy/D.tif]

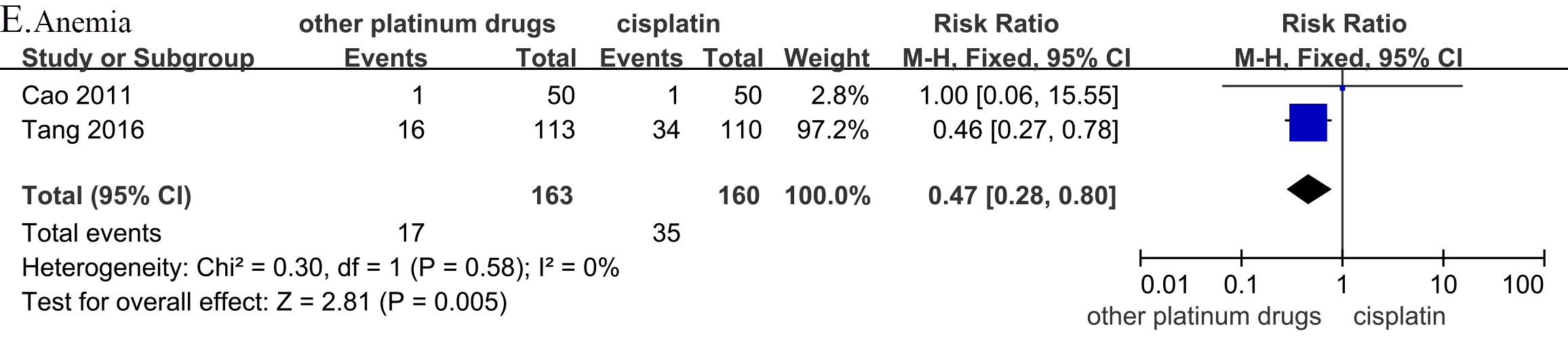

Supplement: Supplementary file 1 — Additional file 1. [file 12885_2022_9712_MOESM1_ESM.zip › Supplementary material/figure/Induction chemotherapy/E .tif]

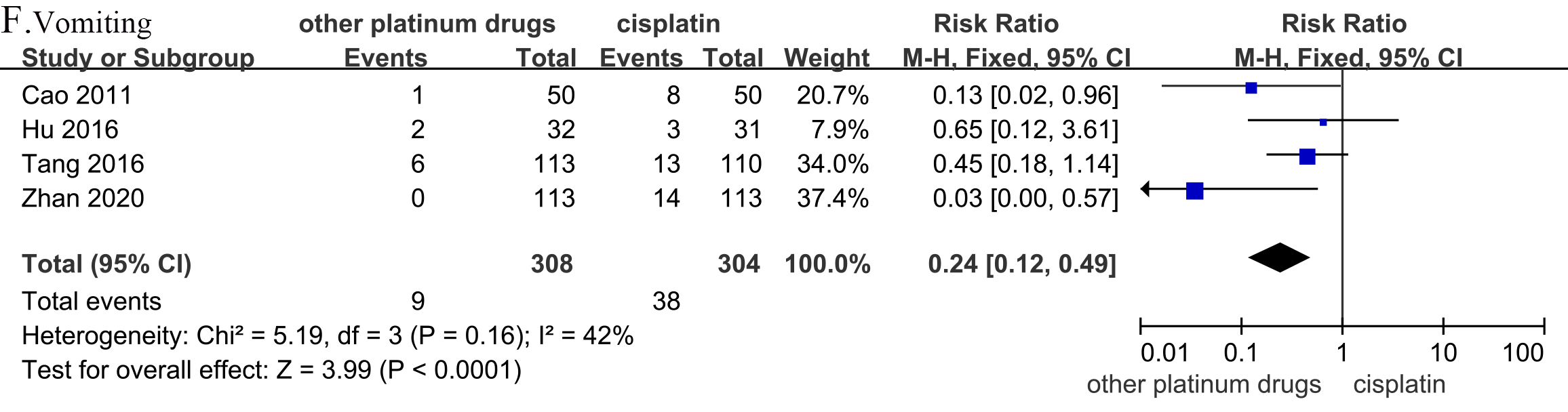

Supplement: Supplementary file 1 — Additional file 1. [file 12885_2022_9712_MOESM1_ESM.zip › Supplementary material/figure/Induction chemotherapy/F.tif]

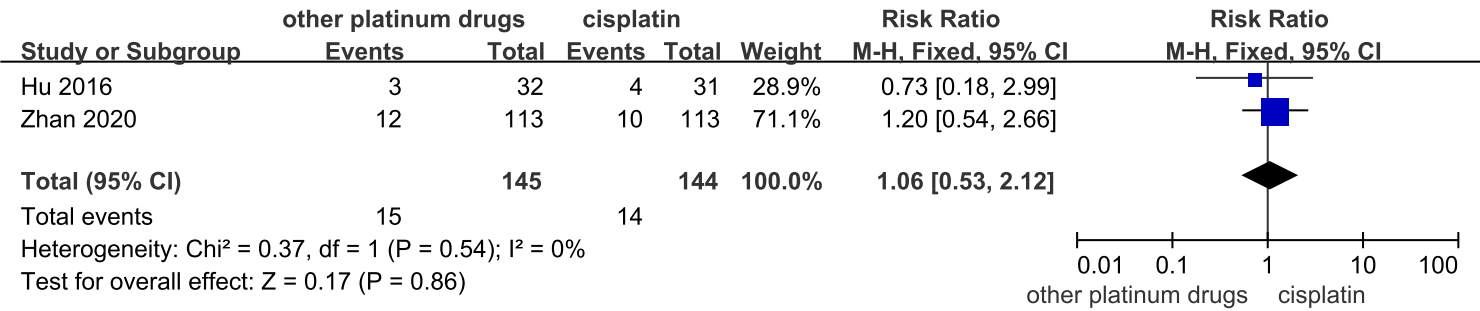

Supplement: Supplementary file 1 — Additional file 1. [file 12885_2022_9712_MOESM1_ESM.zip › Supplementary material/figure/Induction chemotherapy/Leucopenia.pdf]

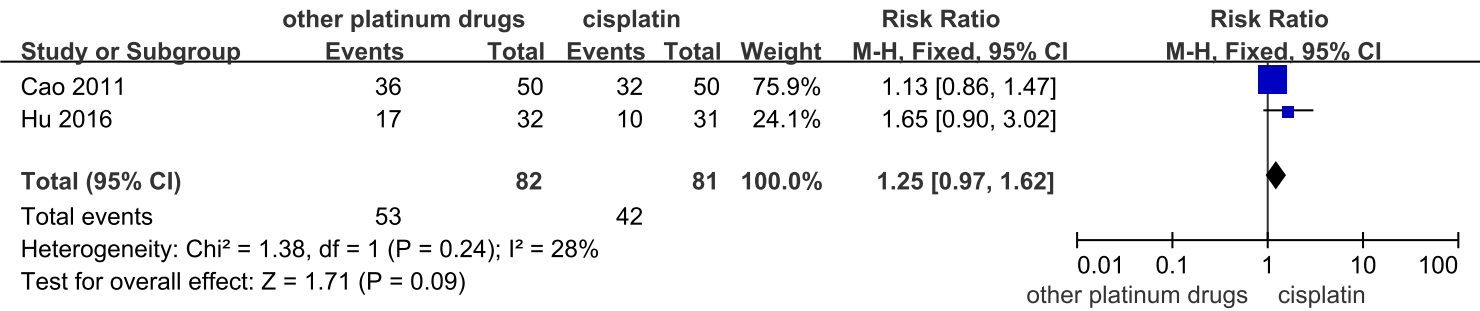

Supplement: Supplementary file 1 — Additional file 1. [file 12885_2022_9712_MOESM1_ESM.zip › Supplementary material/figure/Induction chemotherapy/PR.pdf]

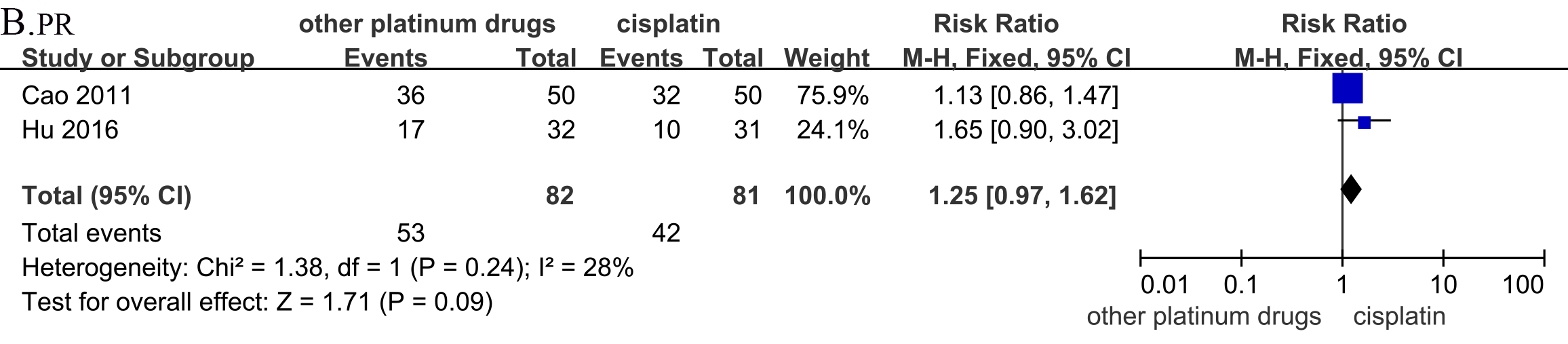

Supplement: Supplementary file 1 — Additional file 1. [file 12885_2022_9712_MOESM1_ESM.zip › Supplementary material/figure/Induction chemotherapy/PR.tif]

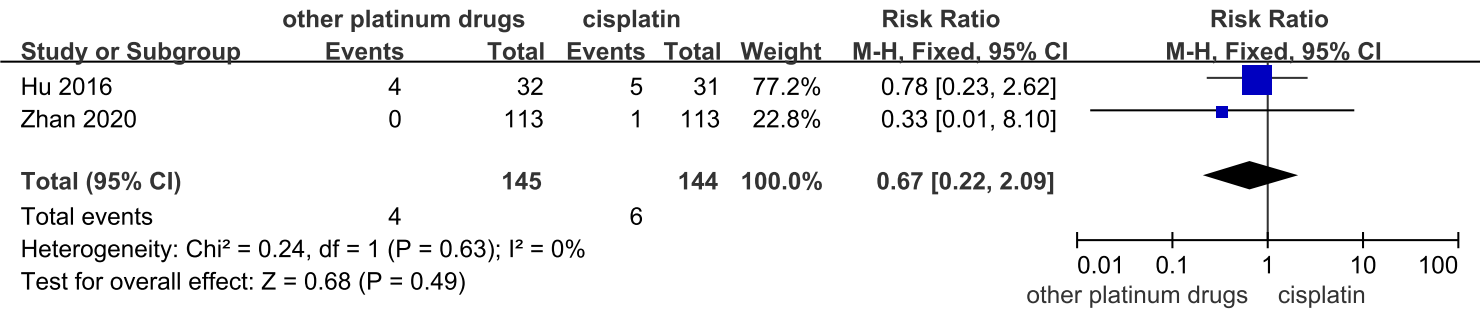

Supplement: Supplementary file 1 — Additional file 1. [file 12885_2022_9712_MOESM1_ESM.zip › Supplementary material/figure/Induction chemotherapy/Thrombocytopenia.pdf]

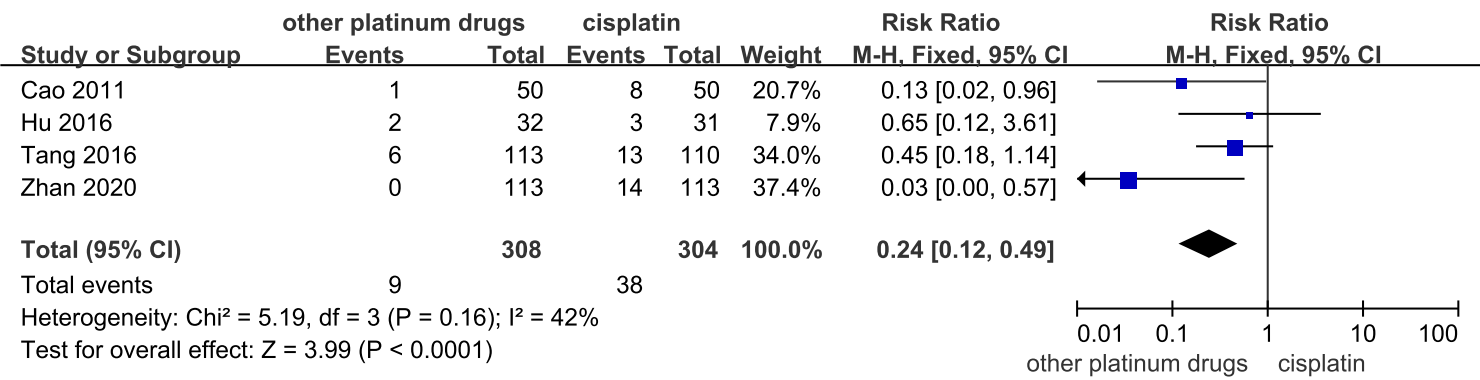

Supplement: Supplementary file 1 — Additional file 1. [file 12885_2022_9712_MOESM1_ESM.zip › Supplementary material/figure/Induction chemotherapy/Vomiting.pdf]

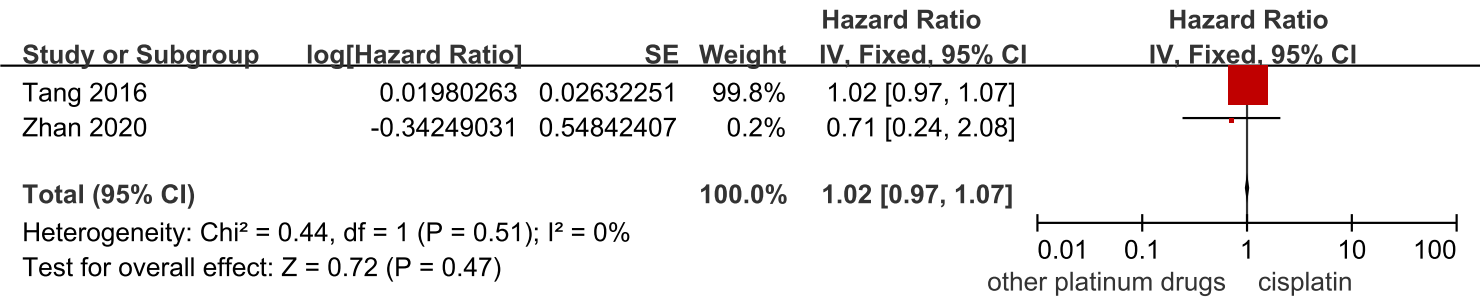

Supplement: Supplementary file 1 — Additional file 1. [file 12885_2022_9712_MOESM1_ESM.zip › Supplementary material/figure/LRF/3LRF.pdf]

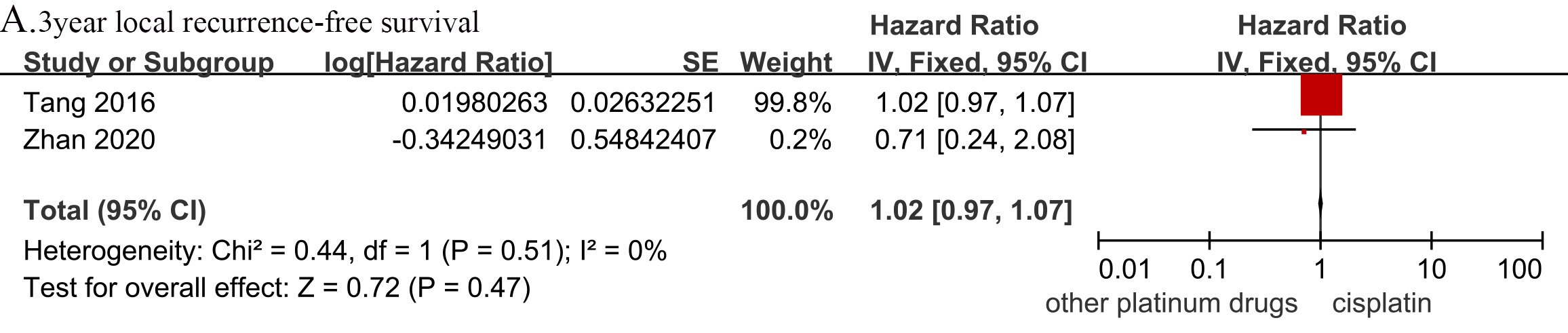

Supplement: Supplementary file 1 — Additional file 1. [file 12885_2022_9712_MOESM1_ESM.zip › Supplementary material/figure/LRF/3LRF.tif]

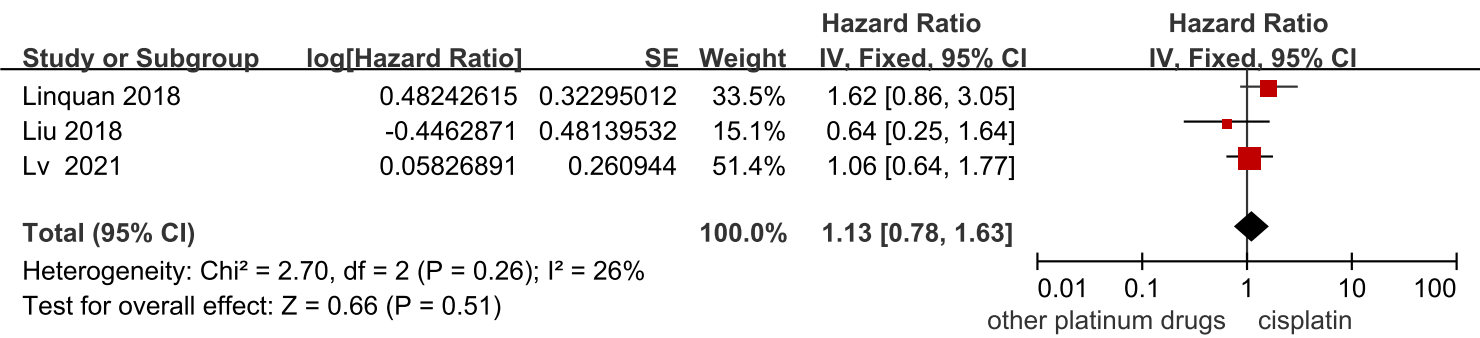

Supplement: Supplementary file 1 — Additional file 1. [file 12885_2022_9712_MOESM1_ESM.zip › Supplementary material/figure/LRF/5LRF.pdf]

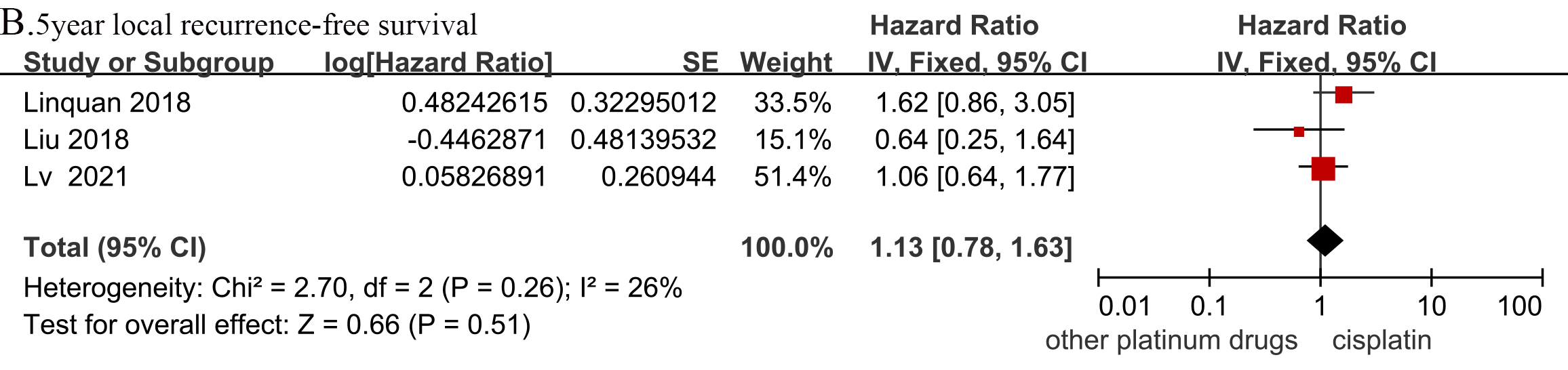

Supplement: Supplementary file 1 — Additional file 1. [file 12885_2022_9712_MOESM1_ESM.zip › Supplementary material/figure/LRF/5LRF.tif]

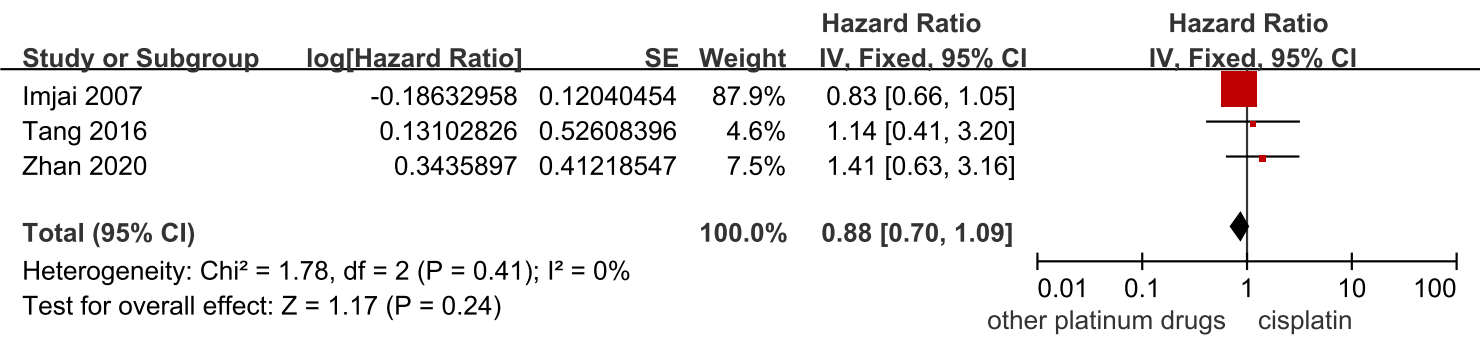

Supplement: Supplementary file 1 — Additional file 1. [file 12885_2022_9712_MOESM1_ESM.zip › Supplementary material/figure/OS/3OS.pdf]

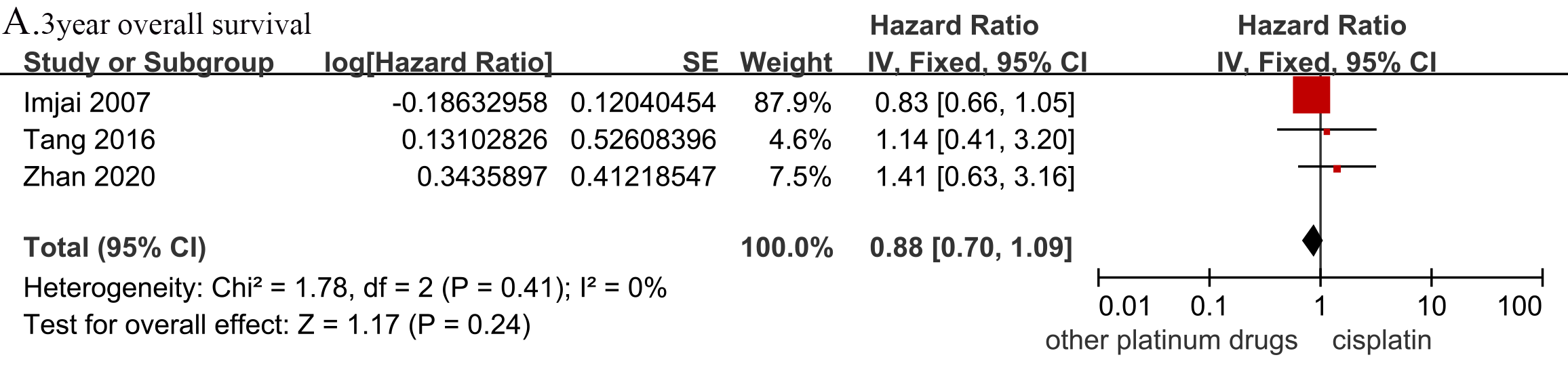

Supplement: Supplementary file 1 — Additional file 1. [file 12885_2022_9712_MOESM1_ESM.zip › Supplementary material/figure/OS/3OS.tif]

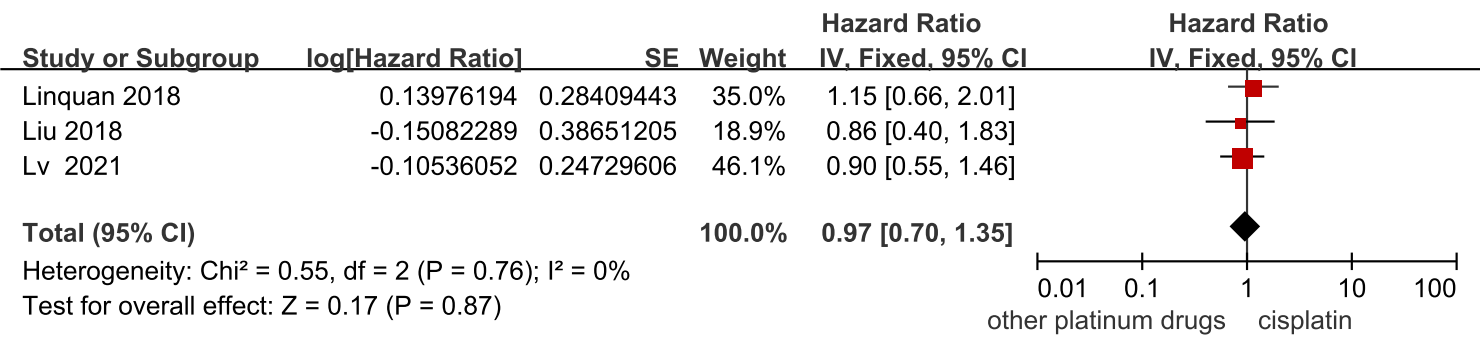

Supplement: Supplementary file 1 — Additional file 1. [file 12885_2022_9712_MOESM1_ESM.zip › Supplementary material/figure/OS/5OS.pdf]

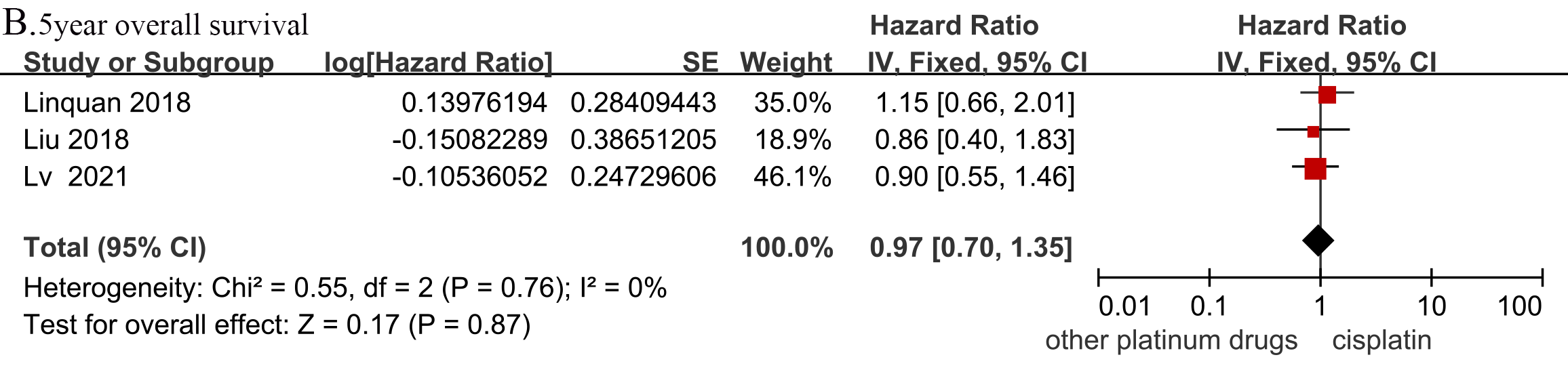

Supplement: Supplementary file 1 — Additional file 1. [file 12885_2022_9712_MOESM1_ESM.zip › Supplementary material/figure/OS/5OS.tif]

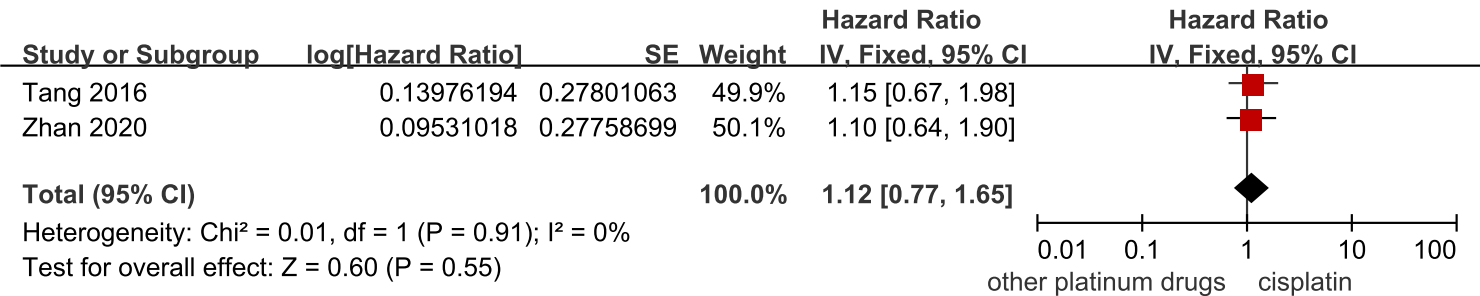

Supplement: Supplementary file 1 — Additional file 1. [file 12885_2022_9712_MOESM1_ESM.zip › Supplementary material/figure/PFS/3PFS.pdf]

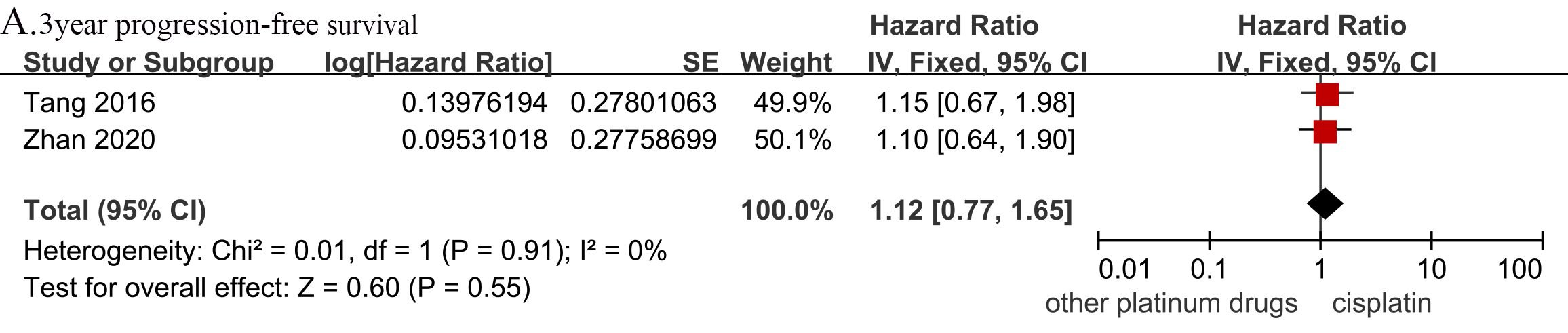

Supplement: Supplementary file 1 — Additional file 1. [file 12885_2022_9712_MOESM1_ESM.zip › Supplementary material/figure/PFS/3PFS.tif]

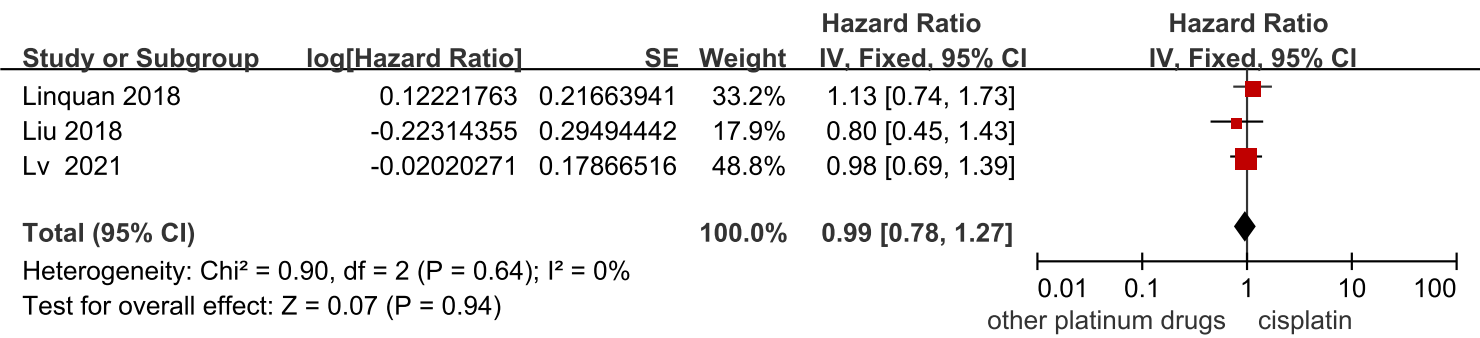

Supplement: Supplementary file 1 — Additional file 1. [file 12885_2022_9712_MOESM1_ESM.zip › Supplementary material/figure/PFS/5PFS.pdf]

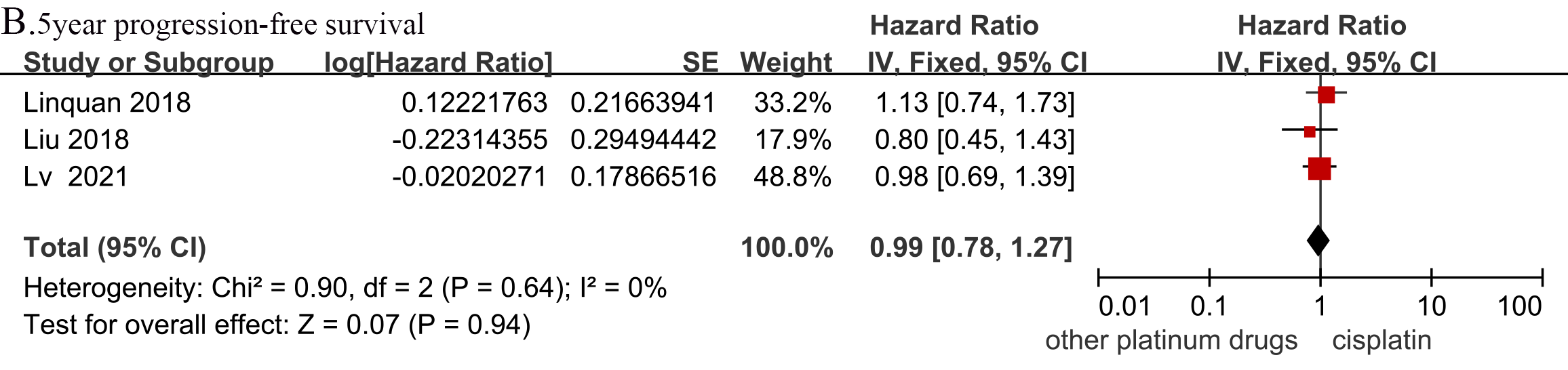

Supplement: Supplementary file 1 — Additional file 1. [file 12885_2022_9712_MOESM1_ESM.zip › Supplementary material/figure/PFS/5PFS.tif]

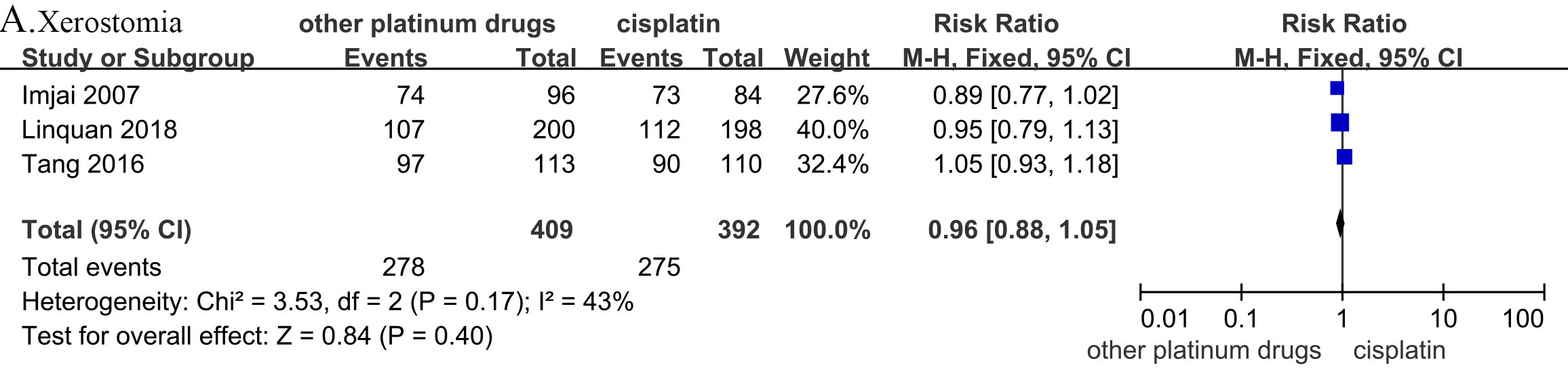

Supplement: Supplementary file 1 — Additional file 1. [file 12885_2022_9712_MOESM1_ESM.zip › Supplementary material/figure/late1-2/A.tif]

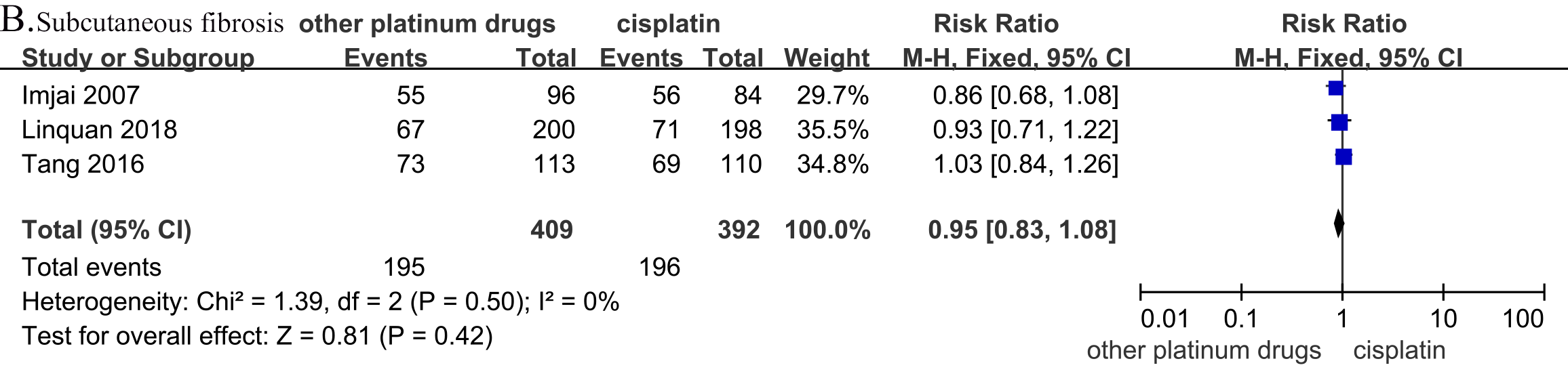

Supplement: Supplementary file 1 — Additional file 1. [file 12885_2022_9712_MOESM1_ESM.zip › Supplementary material/figure/late1-2/B.tif]

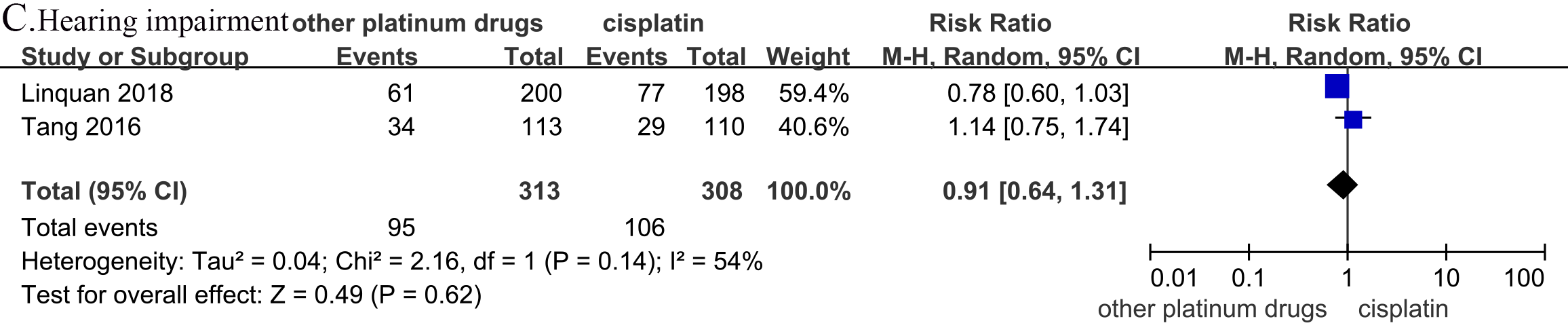

Supplement: Supplementary file 1 — Additional file 1. [file 12885_2022_9712_MOESM1_ESM.zip › Supplementary material/figure/late1-2/C.tif]

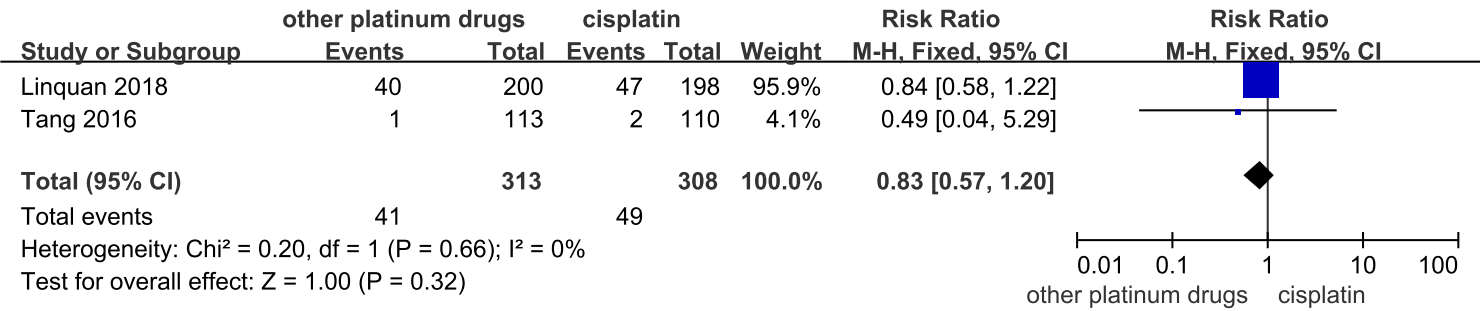

Supplement: Supplementary file 1 — Additional file 1. [file 12885_2022_9712_MOESM1_ESM.zip › Supplementary material/figure/late1-2/Cranial nerve palsy.pdf]

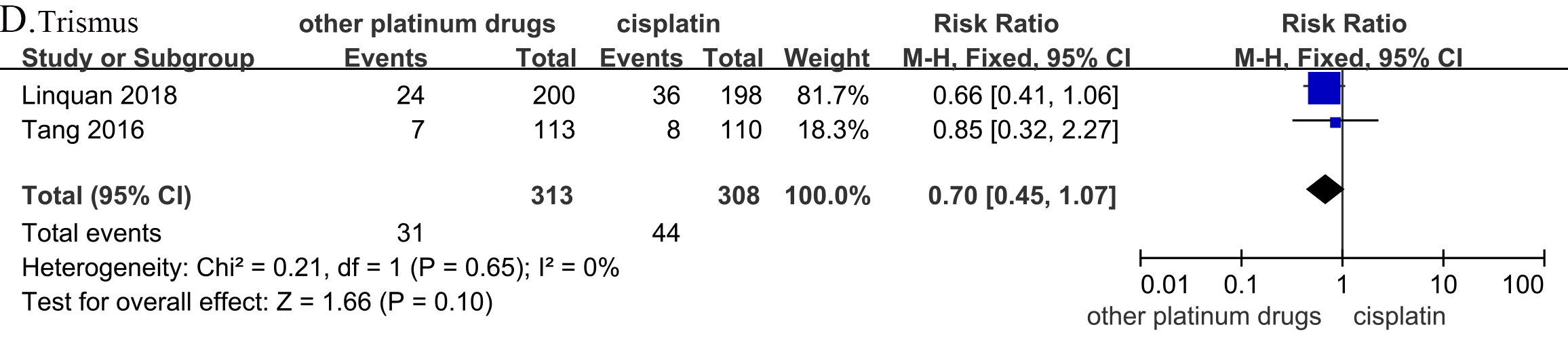

Supplement: Supplementary file 1 — Additional file 1. [file 12885_2022_9712_MOESM1_ESM.zip › Supplementary material/figure/late1-2/D.tif]

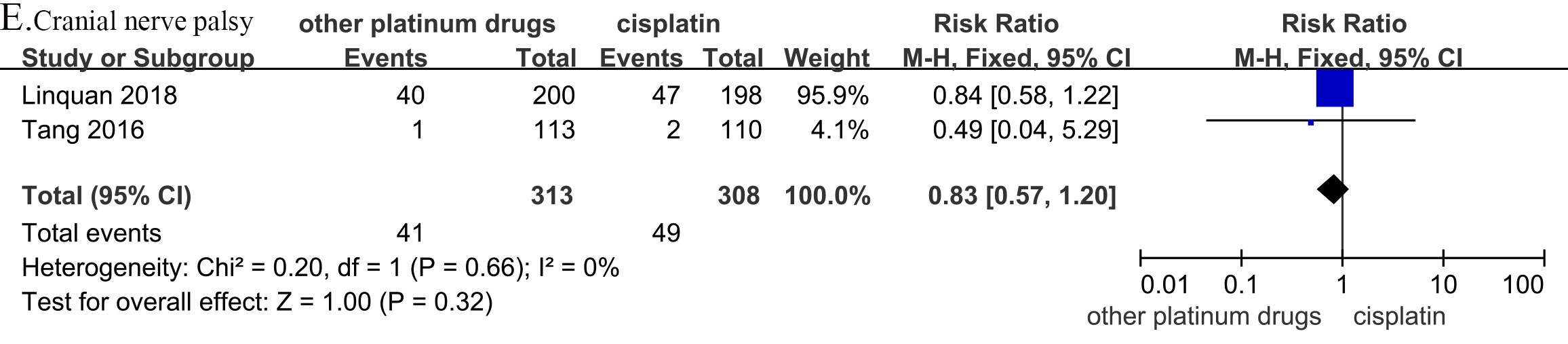

Supplement: Supplementary file 1 — Additional file 1. [file 12885_2022_9712_MOESM1_ESM.zip › Supplementary material/figure/late1-2/E.tif]

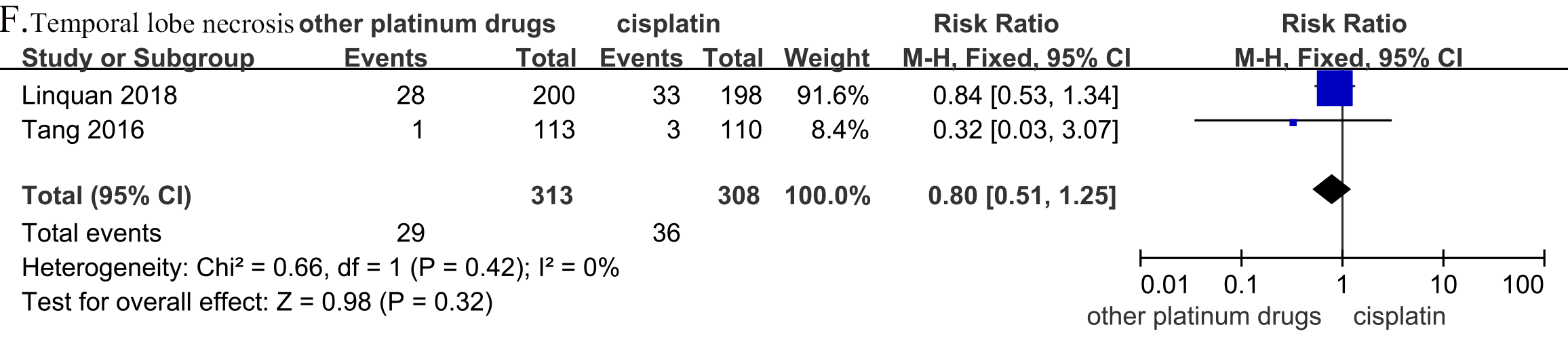

Supplement: Supplementary file 1 — Additional file 1. [file 12885_2022_9712_MOESM1_ESM.zip › Supplementary material/figure/late1-2/F.tif]

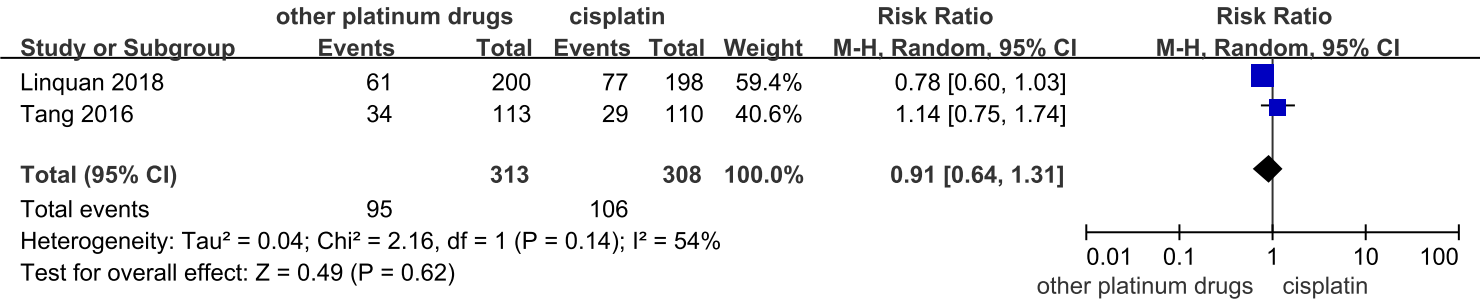

Supplement: Supplementary file 1 — Additional file 1. [file 12885_2022_9712_MOESM1_ESM.zip › Supplementary material/figure/late1-2/Hearing impairment.pdf]

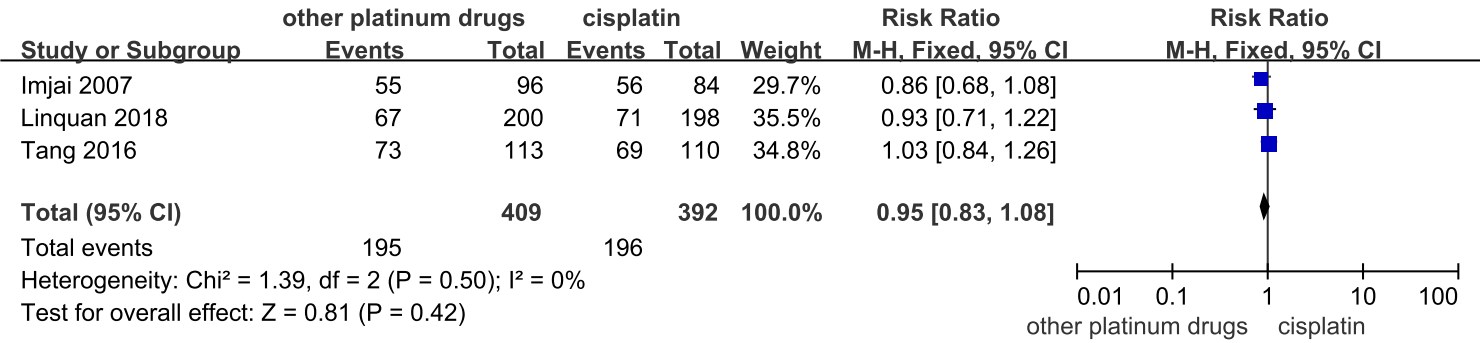

Supplement: Supplementary file 1 — Additional file 1. [file 12885_2022_9712_MOESM1_ESM.zip › Supplementary material/figure/late1-2/Subcutaneous fibrosis.pdf]

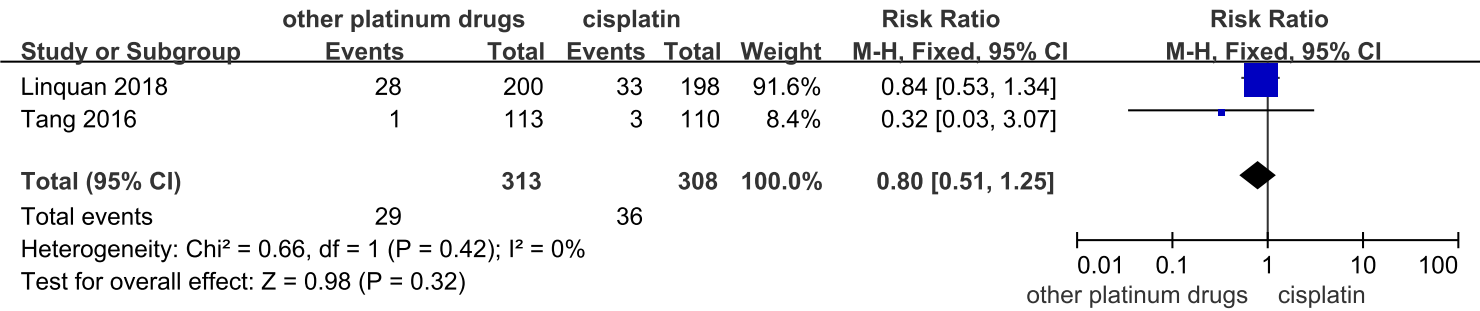

Supplement: Supplementary file 1 — Additional file 1. [file 12885_2022_9712_MOESM1_ESM.zip › Supplementary material/figure/late1-2/Temporal lobe necrosis.pdf]

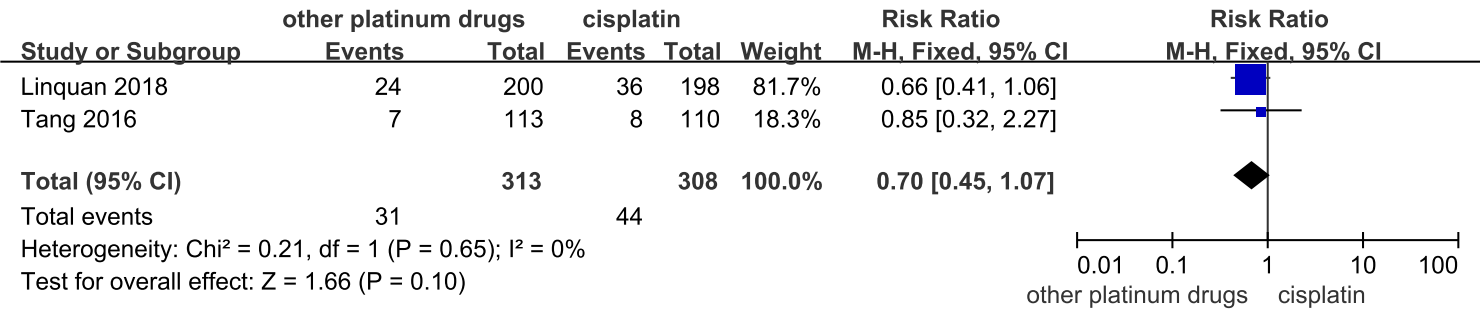

Supplement: Supplementary file 1 — Additional file 1. [file 12885_2022_9712_MOESM1_ESM.zip › Supplementary material/figure/late1-2/Trismus.pdf]

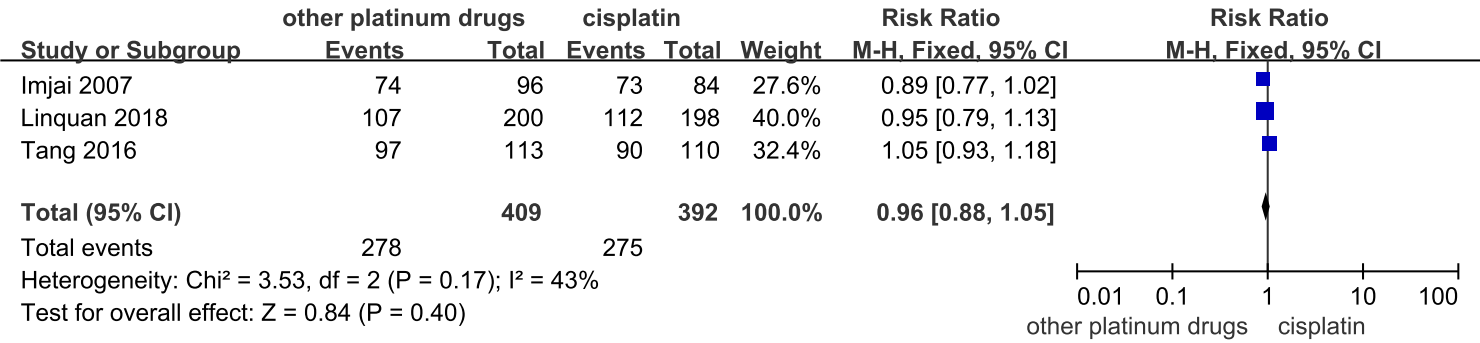

Supplement: Supplementary file 1 — Additional file 1. [file 12885_2022_9712_MOESM1_ESM.zip › Supplementary material/figure/late1-2/Xerostomia.pdf]

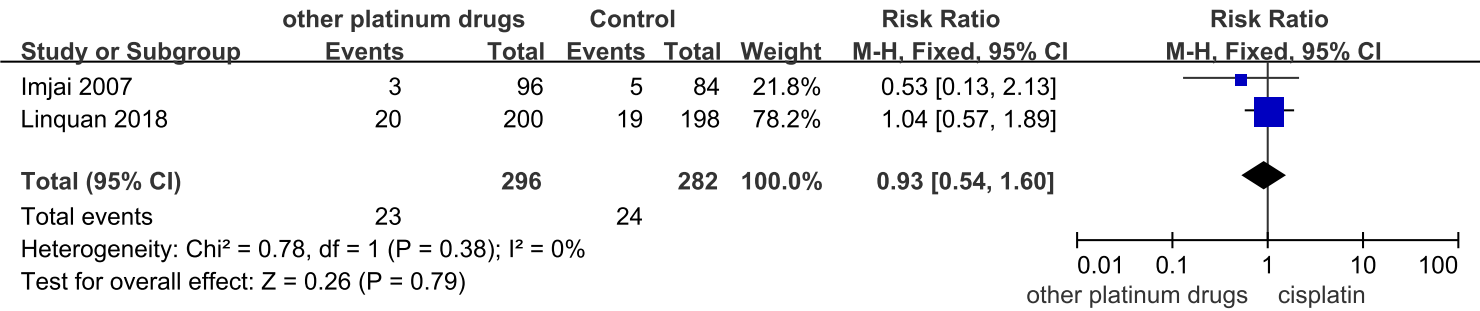

Supplement: Supplementary file 1 — Additional file 1. [file 12885_2022_9712_MOESM1_ESM.zip › Supplementary material/figure/late3-4/Xerostomia.pdf]

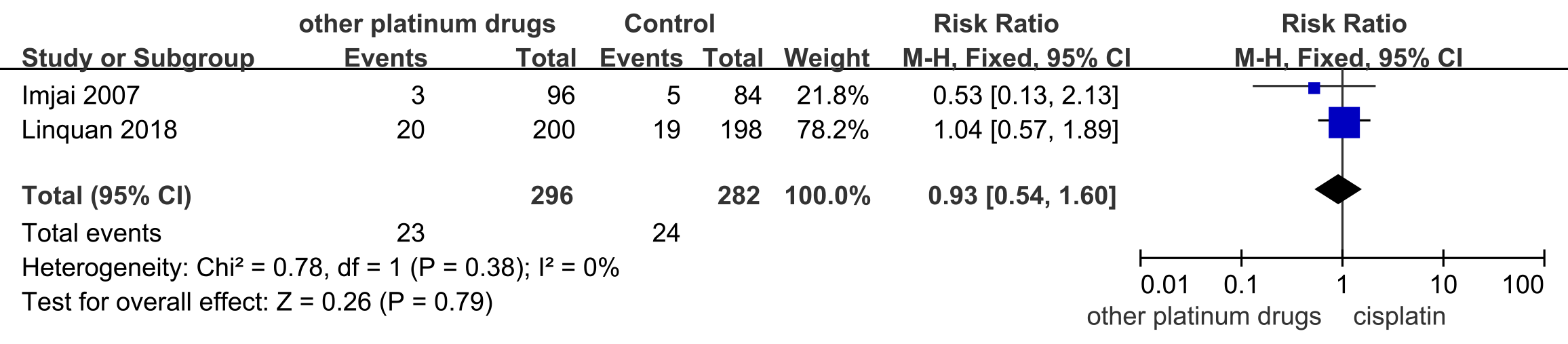

Supplement: Supplementary file 1 — Additional file 1. [file 12885_2022_9712_MOESM1_ESM.zip › Supplementary material/figure/late3-4/Xerostomia.tif]
