# Supplementary material for: Comparing the efficacy and safety of cisplatin and other platinum-based chemotherapies in locally advanced nasopharyngeal carcinoma: a systematic review and meta-analysis
Source: BMC Cancer. 2022 Jun 6;22:616. doi: 10.1186/s12885-022-09712-z (PMC9169397; doi:10.1186/s12885-022-09712-z)
Supplement: Supplementary file 1 — Additional file 1. [file 12885_2022_9712_MOESM1_ESM.zip › Supplementary material/figure/DMFS/▒φ1.doc]

Table 1:Characteristics of the eligible studies

| **Study** | **Journal** | **Year** | **Inclusion period** | **Register** | **Type of study** | **Phase** | **Group** | **No.Patients** | **No.male** | **Mean Age (GroupA\|GroupB)** | **AJCC Stage** | **Median follow-up(year)** | **Jadad score** |
| --- | --- | --- | --- | --- | --- | --- | --- | --- | --- | --- | --- | --- | --- |
| Lv et al. | Lancet Oncol | 2021 | 2013-2015 | ChiCTR-TRC-13003285 | RCT | III | IC+CCRT | 502 | 362 | 43.5\|44 | III–IVB | 75.3 | 3 |
| Tang et al. | American Journal of Cancer Research | 2016 | 2011-2012 | NCT 01479504 | RCT | III | IC+CCRT | 223 | NR | 45.1\|45.3 | III–IV | 35.1 | 3 |
| Linquan et al. | Lancet Oncol ogy | 2018 | 2012-2014 | NCT01540136 | RCT | III | CCRT | 402 | 302 | 44\|45 | II–IVB | 47 | 3 |
| Hu et al. | Journal of Cancer Research and Therapeutics | 2016 | 2014-2015 | NR | RCT | III | IC | 62 | 45 | 50.2\|49.8 | III–IV | NR | 3 |
| Liu et al. | [Journal of Cancer](https://www.baidu.com/link?url=05tdir5S_RNsvJ6Qc1_-0fLwqy-mgeXHk_9aqdiYRsIIXHFplqangiOSHMXYSfn9fbUGAT50Lb0hiYw6iY0aHa&wd=&eqid=92772e410001992f00000006621762dc) | 2018 | 2009-2011 | NR | RE | FE | IC+CCRT | 186 | 119 | NR | II–IVB | 68 | FE |
| Zhan et al. | [Journal of Cancer](https://www.baidu.com/link?url=05tdir5S_RNsvJ6Qc1_-0fLwqy-mgeXHk_9aqdiYRsIIXHFplqangiOSHMXYSfn9fbUGAT50Lb0hiYw6iY0aHa&wd=&eqid=92772e410001992f00000006621762dc) | 2020 | 2012-2017 | NR | RE | FE | IC+CCRT | 226 | 184 | NR | III–IVA | 39.5 | FE |
| Cao et al. | Zhonghua Zhong Liu Za Zhi | 2011 | 2009-2010 | NR | RCT | III | IC | 100 | NR | NR | III–IVA | NR | 3 |
| Imjai et al. | European Journal of Cancer | 2007 | 1999-2004 | NR | RCT | III | CCRT+AC | 206 | 126 | 50\|46 | III–IVB | 26.3 | 3 |
