# Supplementary material for: Comparing the efficacy and safety of cisplatin and other platinum-based chemotherapies in locally advanced nasopharyngeal carcinoma: a systematic review and meta-analysis
Source: BMC Cancer. 2022 Jun 6;22:616. doi: 10.1186/s12885-022-09712-z (PMC9169397; doi:10.1186/s12885-022-09712-z)
Supplement: Supplementary file 1 — Additional file 1. [file 12885_2022_9712_MOESM1_ESM.zip › Supplementary material/retrieval results/1.doc]

新一代铂类对比顺铂治疗局部晚期鼻咽癌的META分析

**疾病类型：** 鼻咽癌

**主题词：**Nasopharyngeal carcinoma

**自由词：**

Carcinoma, Nasopharyngeal

Carcinomas, Nasopharyngeal

Nasopharyngeal Carcinomas

**干预措施：** 奈达铂Nedaplatin， 洛铂lobaplatin 顺铂**Cisplatin**

主题词：**Cisplatin**

**自由词：**

cis-Diamminedichloroplatinum(II)

Platinum Diamminodichloride

Diamminodichloride, Platinum

cis-Platinum

cis Platinum

Dichlorodiammineplatinum

cis-Diamminedichloroplatinum

cis Diamminedichloroplatinum

cis-Dichlorodiammineplatinum(II)

NSC-119875

Platino

Platinol

Biocisplatinum

Platidiam

研究方法：随机对照

**主题词：**随机对照试验andomized controlled trial

**自由词：**randomized controlled trial[Publication Type] OR
randomized[Title/Abstract] OR
placebo[Title/Abstract]

randomized controlled trial

Randomized
placebo

RCT

Pubmed检索式： ((("Nasopharyngeal Carcinoma"[Mesh]) OR ((((Carcinoma, Nasopharyngeal[Title/Abstract]) ) OR (Carcinomas, Nasopharyngeal[Title/Abstract])) OR (Nasopharyngeal Carcinomas[Title/Abstract]))) AND (("Cisplatin"[Mesh]) OR ((((((((((((((cis-Diamminedichloroplatinum(II[Title/Abstract])) OR (Platinum Diamminodichloride[Title/Abstract])) OR (Diamminodichloride, Platinum[Title/Abstract])) OR (cis-Platinum[Title/Abstract])) OR (cis Platinum[Title/Abstract])) OR (Dichlorodiammineplatinum[Title/Abstract])) OR (cis-Diamminedichloroplatinum[Title/Abstract])) OR (cis Diamminedichloroplatinum[Title/Abstract])) OR (cis-Dichlorodiammineplatinum(II[Title/Abstract]))) OR (NSC-119875[Title/Abstract])) OR (Platino[Title/Abstract])) OR (Platinol[Title/Abstract])) OR (Biocisplatinum[Title/Abstract])) OR (Platidiam[Title/Abstract])))) AND (randomized controlled trial[Publication Type] OR randomized[Title/Abstract] OR placebo[Title/Abstract])
